# K-Semistability of cscK Manifolds with Transcendental Cohomology Class

**DOI:** 10.1007/s12220-017-9942-9

**Published:** 2017-10-16

**Authors:** Zakarias Sjöström Dyrefelt

**Affiliations:** 1 Institut de Mathématiques de Toulouse, Université Paul Sabatier, 118 route de Narbonne, 31062 Toulouse Cedex 9, France; 2 Centre de Mathématiques Laurent Schwartz, École Polytechnique, 91128 Palaiseau Cedex, France

**Keywords:** Constant scalar curvature Kähler metric, K-Stability, Energy functional asymptotics, YTD conjecture, 32Q26, 32Q15, 14D06, 53C56, 14F43

## Abstract

We prove that constant scalar curvature Kähler (cscK) manifolds with transcendental cohomology class are K-semistable, naturally generalising the situation for polarised manifolds. Relying on a recent result by R. Berman, T. Darvas and C. Lu regarding properness of the K-energy, it moreover follows that cscK manifolds with discrete automorphism group are uniformly K-stable. As a main step of the proof we establish, in the general Kähler setting, a formula relating the (generalised) Donaldson–Futaki invariant to the asymptotic slope of the K-energy along weak geodesic rays.

## Introduction

In this paper we are interested in questions of stability for constant scalar curvature Kähler (cscK) manifolds with *transcendental*^1^ cohomology class. To this end, let $(X,\omega )$ be a compact Kähler manifold and $\alpha := [\omega ] \in H^{1,1}(X,\mathbb {R})$ the corresponding Kähler class. When $\alpha$ is the first Chern class $c_1(L)$ of some ample line bundle *L* over *X*, such questions are closely related to the Yau–Tian–Donaldson (YTD) conjecture [27, 49, 54]: *A polarised algebraic manifold* (*X*, *L*) *is K-polystable if and only if the polarisation class* $c_1(L)$ *admits a Kähler metric of constant scalar curvature.* This conjecture was recently confirmed in the *Fano case*, i.e. when $L = -K_X$, cf. [16–18, 52]. In this important special case, a cscK metric is nothing but a Kähler–Einstein metric. For general polarised cscK manifolds, the “if” direction of the YTD conjecture was initially proven by Mabuchi in [37], see also [5]. Prior to that, several partial results had been obtained by Donaldson [28] and Stoppa [46], both assuming that $c_1(L)$ contains a cscK metric.

For transcendental classes very little is currently known about the validity of a correspondence between *existence of cscK metrics* and *stability* in the spirit of the YTD conjecture. Moreover, from a differential geometric point of view, there is no special reason to restrict attention to Kähler manifolds with associated integral (or rational) cohomology classes, which are then automatically of the form $\alpha = c_1(L)$ for some ample ($\mathbb {Q}$)-line bundle *L* over *X*. In order to extend the study of stability questions to a transcendental setting, recall that there is an intersection theoretic description of the Donaldson–Futaki invariant, cf. [39, 53]. As first pointed out by Berman [4], a straightforward generalised notion of K-stability in terms of cohomology can thus be defined and a version of the YTD conjecture can be made sense of in this setting. The setup is explained in detail in Sect. 3. Our main goal is to establish the following result:

### Theorem A

Let $(X,\omega )$ be a compact Kähler manifold and let $\alpha := [\omega ] \in H^{1,1}(X,\mathbb {R})$ be the corresponding Kähler class.

(i) If the Mabuchi (*K-energy*) functional is bounded from below in $\alpha$, then $(X,\alpha )$ is *K-semistable* (in the generalised sense of Definition 3.8).
(ii) If the Mabuchi functional is coercive, then $(X,\alpha )$ is uniformly K-stable the generalised sense of Definition 5.4).

For precise definitions we refer to the core of the paper. As an immediate consequence of [6, Theorem 1.1] and the above Theorem A (i) we obtain the following corollary, which is a main motivation for our work (see also Remark 1.2).

### Corollary 1.1

If the Kähler class $\alpha \in H^{1,1}(X,\mathbb {R})$ admits a constant scalar curvature representative, then $(X,\alpha )$ is K-semistable.

The corresponding statement in the case of a polarised manifold was first obtained by Donaldson in [28], as an immediate consequence of the lower bound for the Calabi functional. See also [43, 47] for related work on *slope semistability*. The approach taken in this paper should however be compared to, e.g. [42] and [4, 5, 12, 13], where K-semistability is derived using so called “Kempf–Ness type” formulas. By analogy to the above papers, our proof relies on establishing such formulas valid also for transcendental classes (see Theorems B and C), in particular relating the asymptotic slope of the K-energy along weak geodesic rays to a natural generalisation of the Donaldson–Futaki invariant. This provides a link between K-semistability (resp. uniform K-stability) and boundedness (resp. coercivity) of the Mabuchi functional, key to establishing the stability results of Theorem A.

An underlying theme of the paper is the comparison to the extensively studied case of a polarised manifold, which becomes a “special case” in our setting. Notably, it is then known (see, e.g. [4, 5, 12, 13]) how to establish the sought Kempf–Ness type formulas using *Deligne pairings*; a method employed by Phong–Ross–Sturm in [42] (for further background on the Deligne pairing construction, cf. [30]). Unfortunately, such an approach breaks down in the case of a general Kähler class. In this paper, we circumvent this problem by a pluripotential approach, making use of a certain multivariate variant $\langle \varphi _0, \dots , \varphi _n \rangle _{(\theta _0, \dots \theta _n)}$ of the Monge–Ampère energy functional, which turns out to play a role analogous to that of the Deligne pairing in arguments of the type [42]. The Deligne pairing approach should also be compared to [26, 50] using Bott–Chern forms (see, e.g. 2.4 and [44, Example 5.6]).

### Remark 1.2

(*Yau–Tian–Donaldson conjecture*) Combining Theorem A (ii) with [22, Theorem 2.10] and a very recent result by Berman et al. [8, Theorem 1.2] we in fact further see that cscK manifolds $(X,\alpha )$ with discrete automorphism group are uniformly K-stable. The above thus confirms one direction of the Yau–Tian–Donaldson conjecture, here referring to its natural generalisation to the case of arbitrary compact Kähler manifolds with discrete automorphism group, see Sect. 5.2.

### Generalised K-Semistability

We briefly explain the framework we have in mind. As a starting point, there are natural generalisations of certain key concepts to the transcendental setting, a central notion being that of *test configurations*. First recall that a test configuration for a polarised manifold (*X*, *L*), in the sense of Donaldson, cf. [27], is given in terms of a $\mathbb {C}^*$-equivariant degeneration $(\mathcal {X}, \mathcal {L})$ of (*X*, *L*). It can be seen as an algebrogeometric way of compactifying the product $X \times \mathbb {C}^* \hookrightarrow \mathcal {X}$. Note that test configurations in the sense of Donaldson are now known (at least in the case of Fano manifolds, see [36]) to be equivalent to test configurations in the sense of Tian [49].

As remarked in [4], a straightforward generalisation to the transcendental setting can be given by replacing the line bundles with (1, 1)-cohomology classes. In the polarised setting we would thus consider $(\mathcal {X}, c_1(\mathcal {L}))$ as a “test configuration” for $(X, c_1(L))$, by simply replacing $\mathcal {L}$ and *L* with their respective first Chern classes. The *details* of how to formulate a good definition of such a generalised test configuration have, however, not yet been completely clarified. The definition given in this paper is motivated by a careful comparison to the usual polarised case, where we ensure that a number of basic but convenient tools still hold, cf. Sect. 3. In particular, our notion of K-semistability coincides precisely with the usual one whenever we restrict to the case of an integral class, cf. Proposition 3.14. We will refer to such generalised test configurations as *cohomological*.

#### Definition 1.3

(*Cohomological test configuration*) A cohomological test configuration for $(X, \alpha )$ is a pair $(\mathcal {X},\mathcal {A})$ where $\mathcal {X}$ is a test configuration for *X* (see Definition 3.2) and $\mathcal {A} \in H^{1,1}_{\mathrm {BC}}(\mathcal {X},\mathbb {R})^{\mathbb {C}^*}$ is a $\mathbb {C}^*$-invariant (1, 1)-Bott–Chern cohomology class whose image under the canonical $\mathbb {C}^*$-equivariant isomorphism

$$\begin{aligned} \mathcal {X} {\setminus } \mathcal {X}_0 \simeq X \times (\mathbb {P}^1 {\setminus } \{0\}) \end{aligned}$$

is $p_1^*\alpha$, see (6). Here $p_1: X \times \mathbb {P}^1 \rightarrow X$ denotes the first projection.

#### Remark 1.4

Note that the definition is given directly over $\mathbb {P}^1$ so that we consider the Bott–Chern cohomology on a compact Kähler normal complex space. In the polarised case, defining a test configuration over $\mathbb {C}$ or over $\mathbb {P}^1$ is indeed equivalent, due to the existence of a natural $\mathbb {C}^*$-equivariant compactification over $\mathbb {P}^1$.

In practice, it will be enough to consider the situation when the total space $\mathcal {X}$ is smooth and dominates $X \times \mathbb {P}^1$, with $\mu : \mathcal {X} \rightarrow X \times \mathbb {P}^1$ the corresponding canonical $\mathbb {C}^*$-equivariant bimeromorphic morphism. Moreover, if $(\mathcal {X}, \mathcal {A})$ is a cohomological test configuration for $(X,\alpha )$ with $\mathcal {X}$ as above, then $\mathcal {A}$ is always of the form $\mathcal {A} = \mu ^*p_1^*\alpha + [D]$, for a unique $\mathbb {R}$-divisor *D* supported on the central fibre $\mathcal {X}_0$, cf. Proposition 3.10. A cohomological test configuration can thus be characterised by an $\mathbb {R}$-divisor, clarifying the relationship between the point of view of $\mathbb {R}$-divisors and our cohomological approach to “transcendental K-semistability”.

A straightforward generalisation of the Donaldson–Futaki invariant can be defined based on the intersection theoretic characterisation of [39, 53]. Indeed, we define the *Donaldson–Futaki invariant* associated with a cohomological test configuration $(\mathcal {X},\mathcal {A})$ for $(X,\alpha )$ as the following intersection number

1 $$\begin{aligned} \mathrm {DF}(\mathcal {X}, \mathcal {A}) := \frac{\bar{\mathcal {S}}}{n+1} V^{-1} (\mathcal {A}^{n+1})_{\mathcal {X}} + V^{-1}(K_{\mathcal {X}/\mathbb {P}^1} \cdot \mathcal {A}^n)_{\mathcal {X}}, \end{aligned}$$

computed on the (compact) total space $\mathcal {X}$. Here *V* and $\bar{\mathcal {S}}$ are cohomological constants denoting the Kähler volume and mean scalar curvature of $(X,\alpha )$, respectively.

Finally, we say that $(X,\alpha )$ is *K-semistable* if $\mathrm {DF}(\mathcal {X}, \mathcal {A}) \geqslant 0$ for all cohomological test configurations $(\mathcal {X}, \mathcal {A})$ for $(X,\alpha )$ where the class $\mathcal {A}$ is *relatively Kähler*, i.e. there is a Kähler form $\beta$ on $\mathbb {P}^1$ such that $\mathcal {A} + \pi ^*\beta$ is Kähler on $\mathcal {X}$. Generalised notions of (uniform) K-stability are defined analogously.

### Transcendental Kempf–Ness Type Formulas

As previously stated, a central part of this paper consists in establishing a Kempf–Ness type formula connecting the *Donaldson–Futaki invariant* (in the sense of (1)) with the *asymptotic slope of the K-energy* along certain weak geodesic rays. In fact, we first prove the following result, which is concerned with asymptotics of a certain multivariate analogue of the Monge–Ampère energy, cf. Sect. 2.2 for its definition. It turns out to be very useful for establishing a similar formula for the K-energy (cf. Remark 1.5), but may also be of independent interest.

In what follows, we will work on the level of potentials and refer the reader to Sect. 4 for precise definitions.

#### Theorem B

Let *X* be a compact Kähler manifold of dimension *n* and let $\theta _i$, $0 \leqslant i \leqslant n$, be closed (1, 1)-forms on *X*. Let $(\mathcal {X}_i, \mathcal {A}_i)$ be cohomological test configurations for $(X,\alpha _i)$, where $\alpha _i := [\theta _i] \in H^{1,1}(X,\mathbb {R})$. Then, for each collection of smooth rays $(\varphi _i^t)_{t \geqslant 0}$, $\mathcal {C}^{\infty }$-compatible with $(\mathcal {X}_i, \mathcal {A}_i)$, respectively, the asymptotic slope of the multivariate energy functional $\langle \cdot , \dots , \cdot \rangle := \langle \cdot , \dots , \cdot \rangle _{(\theta _0, \dots , \theta _n)}$ is well defined and satisfies

2 $$\begin{aligned} \frac{\langle \varphi _0^t, \dots , \varphi _n^t \rangle }{t} \longrightarrow (\mathcal {A}_0 \cdot \dots \cdot \mathcal {A}_n) \end{aligned}$$

as $t \rightarrow +\infty$. See Sect. 4.1 for the definition of the above intersection number.

#### Remark 1.5

In the setting of Hermitian line bundles, the above multivariate energy functional naturally appears as the difference (or quotient) of metrics on Deligne pairings. Moreover, note that the above theorem applies to, e.g. Aubin’s $\mathrm {J}$-functional, the Monge–Ampère energy functional $\mathrm {E}$ and its ‘twisted’ version $\mathrm {E}^{\text {Ric}(\omega )}$ but *not* to the K-energy $\mathrm {M}$. Indeed, the expression for $\mathrm {M}(\varphi _t)$ on the form $\langle \varphi _0^t, \dots , \varphi _n^t \rangle _{(\theta _0, \dots , \theta _n)}$ involves the metric $\log (\omega + dd^c\varphi _t)^n$ on the relative canonical bundle $K_{\mathcal {X}/\mathbb {P}^1}$, which blows up close to $\mathcal {X}_0$, cf. Sect. 5. As observed in [13], it is however possible to find functionals of the above form that ‘approximate’ $\mathrm {M}$ in the sense that their asymptotic slopes coincide, up to an explicit correction term that vanishes precisely when the central fibre $\mathcal {X}_0$ is reduced. This is a key observation.

We further remark that such a formula (2) cannot be expected to hold unless the test configurations $(\mathcal {X}_i, \mathcal {A}_i)$ and the rays $(\varphi _i^t)$ are compatible in a certain sense. This is the role of the notion of $\mathcal {C}^{\infty }$-compatibility (as well as the $\mathcal {C}^{1,\bar{1}}$-compatibility used in Theorem C). These notions may seem technical, but in fact mimic the case of a polarised manifold, where the situation is well understood in terms of extension of metrics on line bundles, cf. Sect. 4.1.

As a further important consequence of the above Theorem B we deduce that if $(\mathcal {X}, \mathcal {A})$ is a relatively Kähler cohomological test configuration for $(X,\alpha )$, then for each smooth ray $(\varphi _t)_{t \geqslant 0}$, $\mathcal {C}^{\infty }$-compatible with $(\mathcal {X},\mathcal {A})$, we have the inequality

3 $$\begin{aligned} \lim _{t \rightarrow +\infty } \frac{\mathrm {M}(\varphi _{t})}{t} \leqslant \mathrm {DF}(\mathcal {X}, \mathcal {A}). \end{aligned}$$

This is the content of Theorem  5.1, and should be compared to the discussion in the introduction of [42]. As an important special case, this inequality can be seen to hold in the case of a weak geodesic ray associated with the given test configuration $(\mathcal {X}, \mathcal {A})$, cf. Sect. 4.1 for its construction. The inequality (3) is moreover enough to conclude the proof of Theorem A, as explained in Sect. 5.2.

Using ideas from [13] adapted to the present setting, we may further improve on formula (3) and compute the precise asymptotic slope of the K-energy. In this context, it is natural to consider the *non-Archimedean Mabuchi functional*

$$\begin{aligned} \mathrm {M}^{\mathrm {NA}}(\mathcal {X}, \mathcal {A}) := \mathrm {DF}(\mathcal {X}, \mathcal {A}) + V^{-1}((\mathcal {X}_{0,\mathrm {red}} - \mathcal {X}_0) \cdot \mathcal {A}^n)_{\mathcal {X}}, \end{aligned}$$

cf. [12, 13] for an explanation of the terminology. It is a modification of the Donaldson–Futaki invariant which is *homogeneous under finite base change*, and which satisfies $\mathrm {M}^{\mathrm {NA}}(\mathcal {X}, \mathcal {A}) \leqslant \mathrm {DF}(\mathcal {X}, \mathcal {A})$ with equality precisely when the central fibre $\mathcal {X}_0$ is reduced. We then have the following result, special cases of which have been obtained by previous authors in various different situations and generality.

#### Theorem C

Let $(\mathcal {X}, \mathcal {A})$ be a smooth, relatively Kähler cohomological test configuration for $(X,\alpha )$ dominating $X \times \mathbb {P}^1$. For each subgeodesic ray $(\varphi _t)_{t \geqslant 0}$, $\mathcal {C}^{1,\bar{1}}$-compatible with $(\mathcal {X},\mathcal {A})$, the following limit is well defined and satisfies

$$\begin{aligned} \frac{\mathrm {M}(\varphi _{t})}{t} \longrightarrow \mathrm {M}^{\mathrm {NA}}(\mathcal {X}, \mathcal {A}), \end{aligned}$$

as $t \rightarrow +\infty$. In particular, this result holds for the weak geodesic ray associated with $(\mathcal {X},\mathcal {A})$, constructed in Lemma 4.6.

#### Remark 1.6

When the class $\mathcal {A}$ on $\mathcal {X}$ is merely relatively nef it is possible to obtain similar statements, but this necessitates much more involved arguments. Either way, the above result is more than enough for our purposes here, e.g. for proving the main result, Theorem A.

For polarised manifolds (*X*, *L*) and *smooth* subgeodesic rays $(\varphi _t)_{t \geqslant 0}$, this precise result was proven in [13] using Deligne pairings, as pioneered by Phong–Ross–Sturm in [42] (cf. also Paul–Tian [40, 41]). A formula in the same spirit has also been obtained for the so- called *Ding functional* when *X* is a Fano variety, see [5]. However, it appears as though no version of this result was previously known in the case of non-polarised manifolds.

### Structure of the Paper

In Sect. 2 we fix our notation for energy functionals and subgeodesic rays. In particular, we introduce the multivariate energy functionals $\langle \cdot , \dots , \cdot \rangle _{(\theta _0, \dots , \theta _n)}$, which play a central role in this paper. In Sect. 3 we introduce our generalised notion of *cohomological test configurations* and K-semistability. In the case of a polarised manifold (*X*, *L*), we compare this notion to the usual algebraic one. We also discuss classes of cohomological test configurations for which it suffices to test K-semistability, and establish a number of basic properties. In Sect. 4 we discuss transcendental Kempf–Ness-type formulas and prove Theorem B. This involves introducing natural *compatibility conditions* between a ray $(\varphi _t)$ and a cohomological test configuration $(\mathcal {X}, \mathcal {A})$ for $(X,\alpha )$. As a useful special case, we discuss the weak geodesic ray associated with $(\mathcal {X}, \mathcal {A})$. In Sect. 5 we finally apply Theorem B to yield a weak version of Theorem C, from which we in turn deduce our main result, Theorem A. By an immediate adaptation of techniques from [13] we then compute the precise asymptotic slope of the Mabuchi functional, thus establishing the full Theorem C.

## Preliminaries

### Notation and Basic Definitions

Let *X* be a compact complex manifold of $\mathrm {dim}_{\mathbb {C}}X = n$ equipped with a given Kähler form $\omega$, i.e. a smooth real closed positive (1, 1)-form on *X*. Denote the Kähler class $[\omega ] \in H^{1,1}(X,\mathbb {R})$ by $\alpha$.

In order to fix notation, let $\text {Ric}(\omega ) = -dd^c\log \omega ^n$ be the Ricci curvature form, where $dd^c := \frac{\sqrt{-1}}{2\pi }\partial \bar{\partial }$ is normalised so that $\text {Ric}(\omega )$ represents the first Chern class $c_1(X)$. Its trace

$$\begin{aligned} \mathcal {S}(\omega ) := n \frac{\text {Ric}(\omega ) \wedge \omega ^{n-1}}{\omega ^n} \end{aligned}$$

is the scalar curvature of $\omega$. The mean scalar curvature is the cohomological constant given by

4 $$\begin{aligned} \bar{\mathcal {S}} := V^{-1} \int _X \mathcal {S}(\omega ) \; \omega ^n = n\frac{\int _X c_1(X) \cdot \alpha ^{n-1}}{ \int _X \alpha ^n} := n \frac{(c_1(X) \cdot \alpha ^{n-1})_X}{( \alpha ^n)_X}, \end{aligned}$$

where $V := \int _X \omega ^n := ( \alpha ^n)_X$ is the Kähler volume. We say that $\omega$ is a constant scalar curvature Kähler (cscK) metric^2^ if $S(\omega )$ is constant (equal to $\bar{\mathcal {S}}$) on X.

Throughout the paper we work on the level of potentials, using the notation of quasi-plurisubharmonic (quasi-psh) functions. To this end, we let $\theta$ be a closed (1, 1)-form on *X* and denote, as usual, by $\mathrm {PSH}(X,\theta )$ the space of $\theta$-psh functions $\varphi$ on *X*, i.e. the set of functions that can be locally written as the sum of a smooth and a plurisubharmonic function, and such that

$$\begin{aligned} \theta _{\varphi } := \theta + dd^c\varphi \geqslant 0 \end{aligned}$$

in the weak sense of currents. In particular, if $\omega$ is our fixed Kähler form on *X*, then we write

$$\begin{aligned} \mathcal {H}_{\omega } := \{ \varphi \in \mathcal {C}^{\infty }(X) : \omega _{\varphi } := \omega + dd^c\varphi > 0 \} \subset \mathrm {PSH}(X,\omega ) \end{aligned}$$

for the space of Kähler potentials on *X*. As a subset of $\mathcal {C}^{\infty }(X)$ it is convex and consists of strictly $\omega$-psh functions. It has been extensively studied (for background we refer the reader to, e.g. [10] and references therein).

Recall that a $\theta$-psh function is always upper semi-continuous (usc) on *X*, thus bounded from above by compactness. Moreover, if $\varphi _i \in \mathrm {PSH}(X,\theta ) \cap L^{\infty }_{\mathrm {loc}}$, $1 \leqslant i \leqslant p \leqslant n$, it follows from the work of Bedford–Taylor [2, 3] that we can give meaning to the product $\bigwedge _{i = 1}^p (\theta + dd^c\varphi _i)$, which then defines a closed positive (*p*, *p*)-current on *X*. As usual, we then define the *Monge–Ampère measure* as the following probability measure, given by the top wedge product

$$\begin{aligned} \mathrm {MA}(\varphi ) := V^{-1}(\omega + dd^c\varphi )^{n}. \end{aligned}$$

### Energy Functionals and a Deligne Functional Formalism

We now introduce the notation for energy functionals that we will use. Let $\varphi _i \in \mathrm {PSH}(X,\omega ) \cap L^{\infty }_{\mathrm {loc}}$. The *Monge–Ampère energy functional* (or *Aubin–Mabuchi functional*) $\mathrm {E} := \mathrm {E}_{\omega }$ is defined by

$$\begin{aligned} \mathrm {E}(\varphi ) := \frac{1}{n+1}\sum _{j=0}^n V^{-1} \int _X \varphi (\omega + dd^c\varphi )^{n-j} \wedge \omega ^j. \end{aligned}$$

Similarly, if $\theta$ is any closed (1, 1)-form, we define a functional $\mathrm {E}^{\theta } := \mathrm {E}_{\omega }^{\theta }$ by

$$\begin{aligned} \mathrm {E}^{\theta }(\varphi ) := \sum _{j=0}^{n-1} V^{-1} \int _X \varphi (\omega + dd^c\varphi )^{n-j-1} \wedge \omega ^j \wedge \theta , \end{aligned}$$

and we will also have use for the Aubin $\mathrm {J}$-functional $\mathrm {J}: \mathrm {PSH}(X,\omega ) \cap L^{\infty }_{\mathrm {loc}} \rightarrow \mathbb {R}_{\geqslant 0}$ defined by

$$\begin{aligned} \mathrm {J}(\varphi ) := V^{-1} \int _X \varphi \; \omega ^n - \mathrm {E}(\varphi ), \end{aligned}$$

whose asymptotic slope along geodesic rays is comparable with the *minimum norm* of a test configuration (see [12, 24]).

More generally, it is possible to define a natural *multivariate* version of the Monge–Ampère energy, of which all of the above functionals are special cases. The construction builds on that of the *Deligne pairing*, which is a powerful and general technique from algebraic geometry that we here apply to our specific setting in Kähler geometry. We refer the interested reader to [30, 38, 55] for a general treatment of Deligne pairings, as well as to [5, 13, 42] for more recent applications related to K-stability. Now let $\theta _0, \dots , \theta _n$ be closed (1, 1)-forms on *X*. Motivated by corresponding properties for the *Deligne pairing* (cf., e.g. [5, 30] for background) we would like to consider a *functional*$\langle \cdot , \dots , \cdot \rangle _{(\theta _0,\dots ,\theta _n)}$ on the space $\mathrm {PSH}(X,\theta _0) \cap L^{\infty }_{\mathrm {loc}} \times \dots \times \mathrm {PSH}(X,\theta _n) \cap L^{\infty }_{\mathrm {loc}}$ ($n + 1$ times) that is

- symmetric, i.e. for any permutation $\sigma$ of the set $\{0,1, \dots ,n\}$, we have
  $$\begin{aligned} \langle \varphi _{\sigma (0)}, \ldots , \varphi _{\sigma (n)} \rangle _{(\theta _{\sigma (0)}, \ldots , \theta _{\sigma (n)})} = \langle \varphi _0, \ldots , \varphi _n \rangle _{(\theta _0,\dots ,\theta _n)}. \end{aligned}$$
- if $\varphi _0'$ is another $\theta _i$-psh function in $\mathrm {PSH}(X,\theta ) \cap L^{\infty }_{\mathrm {loc}}$, then we have a ‘change of function’ property
  $$\begin{aligned}&\langle \varphi _0', \varphi _1 \dots , \varphi _n \rangle - \langle \varphi _0, \varphi _1 \dots , \varphi _n \rangle \\&\quad =\int _X (\varphi _0' - \varphi _0) \; (\omega _1 + dd^c\varphi _1) \wedge \dots \wedge (\omega _n + dd^c\varphi _n). \end{aligned}$$

Demanding that the above properties hold necessarily leads to the following definition of *Deligne functionals*, that will provide a useful terminology for this paper.

#### Definition 2.1

Let $\theta _0, \dots , \theta _n$ be closed (1, 1)-forms on *X*. Define a *multivariate energy functional*$\langle \cdot , \dots , \cdot \rangle _{(\theta _0,\dots ,\theta _n)}$ on the space $\mathrm {PSH}(X,\theta _0) \cap L^{\infty }_{\mathrm {loc}} \times \dots \times \mathrm {PSH}(X,\theta _n) \cap L^{\infty }_{\mathrm {loc}}$ ($n + 1$ *times)* by

$$\begin{aligned}&\langle \varphi _0, \dots , \varphi _n \rangle _{(\theta _0,\dots ,\theta _n)} := \int _X \varphi _0 \; (\theta _1 + dd^c\varphi _1) \wedge \dots \wedge (\theta _n + dd^c\varphi _n)\\&\quad + \int _X \varphi _1 \; \theta _0 \wedge (\theta _2 + dd^c\varphi _2) \wedge \dots \wedge (\theta + dd^c\varphi _n) + \dots + \int _X \varphi _n \; \theta _0 \wedge \dots \wedge \theta _{n-1}. \end{aligned}$$

#### Remark 2.2

The multivariate energy functional $\langle \cdot , \dots , \cdot \rangle _{(\theta _0,\dots ,\theta _n)}$ can also be defined on $\mathcal {C}^{\infty }(X) \times \dots \times \mathcal {C}^{\infty }(X)$ by the same formula. In Sects. 4 and 5 it will be interesting to consider both the smooth case and the case of locally bounded $\theta _i$-psh functions.

Using integration by parts one can check that this functional is indeed symmetric.

#### Proposition 2.3

The functional $\langle \cdot , \dots , \cdot \rangle _{(\theta _0,\dots ,\theta _n)}$ is symmetric.

#### Proof

Since every permutation is a composition of transpositions it suffices to check the sought symmetry property for transpositions $\sigma := \sigma _{j,k}$ exchanging the position of $j,k \in \{0,1,\dots ,n\}$. Suppose for simplicity of notation that $j < k$ and write $\theta _i^t := \theta _i + dd^c\varphi _i$. A straightforward computation then yields

$$\begin{aligned}&\langle \varphi _{0}, \dots ,\varphi _j, \varphi _k, \dots \varphi _{n} \rangle _{(\theta _{0}, \dots , \theta _j, \theta _k, \dots \theta _{n})} - \langle \varphi _{0}, \dots ,\varphi _k, \varphi _j, \dots \varphi _{n} \rangle _{(\theta _{0}, \dots , \theta _k, \theta _j, \dots \theta _{n})} \\&\quad = \int _X \varphi _jdd^c\varphi _k \wedge \Theta _{j,k} - \int _X \varphi _kdd^c\varphi _j \wedge \Theta _{j,k} = 0, \end{aligned}$$

where in the last step we used integration by parts and write

$$\begin{aligned} \Theta _{j,k} := \theta _0 \wedge \dots \wedge \theta _{j-1} \wedge \theta _{j+1}^t \wedge \dots \theta _{k-1}^t \wedge \theta _{k+1}^t \wedge \theta _n^t, \end{aligned}$$

(with factors $\theta _j$ and $\theta _k^t$ omitted). The case $j > k$ follows in the exact same way, with obvious modifications to the above proof. $\square$

#### Example 2.4

As previously remarked, note that the above functionals can be written using the Deligne functional formalism. Indeed, if $\theta$ is a closed (1, 1)-form on *X*, $\omega$ is a Kähler form on *X* and $\varphi$ is an $\omega$-psh function on *X*, then

$$\begin{aligned} \mathrm {E}(\varphi ) = \frac{1}{n+1} V^{-1} \langle \varphi , \dots , \varphi \rangle _{(\omega , \dots , \omega )} \;, \; \; \mathrm {E}^{\theta }(\varphi ) = V^{-1} \langle 0,\varphi , \dots , \varphi \rangle _{(\theta , \omega , \dots , \omega )} \end{aligned}$$

and

$$\begin{aligned} \mathrm {J}(\varphi ) = V^{-1} \langle \varphi ,0, \dots , 0 \rangle _{(\omega , \dots , \omega )} - \mathrm {E}(\varphi ). \end{aligned}$$

Compare also [44, Example 5.6] on Bott–Chern forms.

### Subgeodesic Rays

Let $(\varphi _t)_{t \geqslant 0} \subset \mathrm {PSH}(X,\omega )$ be a ray of $\omega$-psh functions. Following a useful point of view of Donaldson [27] and Semmes [45], there is a basic correspondence between the family $(\varphi _t)_{t \geqslant 0}$ and an associated $S^1$-invariant function $\Phi$ on $X \times \bar{\Delta }^*$, where $\bar{\Delta }^*\subset \mathbb {C}$ denotes the punctured unit disc. We denote by $\tau$ the coordinate on $\Delta$. Explicitly, the correspondence is given by

$$\begin{aligned} \Phi (x,e^{-t+is}) = \varphi ^t(x), \end{aligned}$$

where the sign is chosen so that $t \rightarrow +\infty$ corresponds to $\tau := e^{-t+is} \rightarrow 0$. The function $\Phi$ restricted to a fibre $X \times \{\tau \}$ thus corresponds precisely to $\varphi _t$ on *X*. In the direction of the fibres we thus have $p_1^*\omega + dd^c_{x}\Phi \geqslant 0$ (in the sense of currents, letting $p_1: X \times \Delta \rightarrow X$ denote the first projection).

We will use the following standard terminology, motivated by the extensive study of (weak) geodesics in the space $\mathcal {H}$, see, e.g. [9, 15, 20, 27, 45].

#### Definition 2.5

We say that $(\varphi _t)_{t \geqslant 0}$ is a *subgeodesic ray* if the associated $S^1$-invariant function $\Phi$ on $X \times \bar{\Delta }^*$ is $p_1^*\omega$-psh. Furthermore, a locally bounded family of functions $(\varphi _t)_{t \geqslant 0}$ in $\mathrm {PSH}(X,\omega )$ is said to be a *weak geodesic ray* if the associated $S^1$-invariant function $\Phi \in \mathrm {PSH}(X \times \bar{\Delta }^*, p_1^*\omega )$ satisfies

$$\begin{aligned} \left( p_1^*\omega + dd^c_{(x,\tau )}\Phi \right) ^{n+1} = 0 \end{aligned}$$

on $$X \times \Delta ^*$$.

#### Definition 2.6

Viewing the family $(\varphi _t)_{t \geqslant 0}$ as a map $(0,+\infty ) \rightarrow \mathrm {PSH}(X,\omega )$, we say that $(\varphi ^t)_{t \geqslant 0}$ is continuous (resp. locally bounded, smooth) if the corresponding $S^1$-invariant function $\Phi$ is continuous (resp. locally bounded, smooth).

The existence of geodesics with bounded Laplacian was proven by Chen [15] with complements by Blocki [9], see also, e.g. [20, 21]. We will refer to such a geodesic as being $\mathcal {C}^{1,\bar{1}}$-*regular*, cf. Lemma 4.6.

#### Definition 2.7

We say that a function $\varphi$ is $\mathcal {C}^{1,\bar{1}}$*-regular* if $dd^c\varphi \in L^{\infty }_{\mathrm {loc}}$, and we set $\mathcal {H}^{1,\bar{1}}:= \mathrm {PSH}(X,\omega ) \cap \mathcal {C}^{1,\bar{1}}$.

Recall that a $\mathcal {C}^{1,\bar{1}}$-regular function is automatically $\mathcal {C}^{1,a}$-regular for all $0< a < 1$. On the other hand, this condition is weaker than $\mathcal {C}^{1,1}$-regularity (i.e. bounded real Hessian).

### Second-Order Variation of Deligne Functionals

We have the following identity for the second-order variations of the multivariate energy functional $\langle \cdot , \dots , \cdot \rangle _{(\theta _0, \dots , \theta _n)}$.

#### Proposition 2.8

Let $\theta _0, \dots , \theta _n$ be closed (1, 1)-forms on *X* and let $(\varphi _i^t)_{t \geqslant 0}$ be a smooth ray of smooth functions. Let $\tau := e^{-t + is}$ and consider the reparametrised ray $(\varphi _i^{\tau })_{\tau \in \bar{\Delta }^*}$. Denoting by $\Phi _i$ the corresponding $S^1$-invariant function on $X \times \Delta ^*$, we have

$$\begin{aligned} dd^c_{\tau } \langle \varphi _0^{\tau }, \dots , \varphi _n^{\tau } \rangle _{(\theta _0, \dots , \theta _n)} = \int _X (p_1^*\theta _0 + dd^c_{(x,\tau )}\Phi _0) \wedge \dots \wedge (p_1^*\theta _n + dd^c_{(x,\tau )}\Phi _n) \end{aligned}$$

where $\int _X$ denotes fibre integration, i.e. pushforward of currents.

#### Proof

The result follows from a computation relying on integration by parts and is an immediate adaptation of, for instance, [7, Proposition 6.2]. $\square$

As a particular case of the above, we obtain the familiar formulas for the second-order variation of *E* and $E^{\theta }$, given by

$$\begin{aligned} dd^c_{\tau }\mathrm {E}(\varphi _{\tau }) = \frac{1}{n + 1} V^{-1} \int _X (p_1^*\omega + dd_{(x,\tau )}^c\Phi )^{n+1} \end{aligned}$$

and

$$\begin{aligned} dd^c_{\tau }\mathrm {E}^{\theta }(\varphi _{\tau }) = V^{-1} \int _X (p_1^*\omega + dd_{(x,\tau )}^c\Phi )^{n} \wedge \theta \end{aligned}$$,

respectively. In particular, note that $\mathrm {E}(\varphi _{\tau }) := \mathrm {E} \circ \Phi$ is a subharmonic function on $\bar{\Delta }^*$, whenever $(\varphi _{\tau })$ is a subgeodesic. The function $t \mapsto \mathrm {E}(\varphi _{\tau })$ is *affine* along weak geodesics, and *convex* along subgeodesics.

### The K-Energy and the Chen–Tian Formula

Let $\omega$ be a Kähler form on *X* and consider any path $(\varphi _t)_{t \geqslant 0}$ in the space $\mathcal {H}$ of Kähler potentials on *X*. The *Mabuchi functional* (or *K-energy*) $\mathrm {M}: \mathcal {H} \rightarrow \mathbb {R}$ is then defined by its Euler–Lagrange equation

$$\begin{aligned} \frac{d}{dt} \mathrm {M}(\varphi _t) = V^{-1} \int _X \dot{\varphi }_t(\mathcal {S}(\omega _{\varphi _t}) - \bar{\mathcal {S}})\; \omega _{\varphi _t}^n. \end{aligned}$$

It is indeed independent of the path chosen, and the critical points of the Mabuchi functional are precisely the cscK metrics, when they exist. By *the Chen–Tian formula* [14] it is possible to write the Mabuchi functional as a sum of an “energy” and an “entropy” part. More precisely, with our normalisations we have

5 $$\begin{aligned} \mathrm {M}(\varphi ) = \left( \bar{\mathcal {S}} \mathrm {E}(\varphi ) - \mathrm {E}^{\text {Ric}(\omega )}(\varphi )\right) + V^{-1} \int _X \log \left( \frac{(\omega + dd^c\varphi )^n}{\omega ^n} \right) (\omega + dd^c\varphi )^n, \end{aligned}$$

where the latter term is the *relative entropy* of the probability measure $\mu := \omega _{\varphi }^n/V$ with respect to the reference measure $\mu _0 := \omega ^n/V$. Recall that the entropy takes values in $[0, +\infty ]$ and is finite if $\mu /\mu _0$ is bounded. It can be seen to be always lower semi-continuous (lsc) in $\mu$.

Following Chen [14] (using the formula (5)) we will often work with the extension $\mathrm {M}:\mathcal {H}^{1,\bar{1}} \rightarrow \mathbb {R}$ of the Mabuchi functional to the space of $\omega$-psh functions with bounded Laplacian. This is a natural setting to consider, since weak geodesic rays with bounded Laplacian are known to always exist, cf. [9, 15, 20, 21] as well as Lemma 4.6.

For later use, we also state the following definition.

#### Definition 2.9

The Mabuchi K-energy functional is said to be *coercive* if there are constants $\delta , C > 0$ such that

$$\begin{aligned} \mathrm {M}(\varphi ) \geqslant \delta \mathrm {J}(\varphi ) - C \end{aligned}$$

for all $$\varphi \in \mathcal {H}$$.

We further recall that the Mabuchi functional is convex along weak geodesic rays, as was recently established by [6], see also [19]. As a consequence of this convexity, the Mabuchi functional is bounded from below (in the given Kähler class) whenever $\alpha$ contains a cscK metric, see [29, 35] for a proof in the polarised case and [6] for the general Kähler setting.

## Cohomological Test Configurations and K-Semistability

In this section we introduce a natural generalised notion of test configurations and K-semistability of $(X,\alpha )$ that has meaning even when the class $\alpha \in H^{1,1}(X,\mathbb {R})$ is non-integral (or non-rational), i.e. when $\alpha$ is not necessarily of the form $c_1(L)$ for some ample ($\mathbb {Q}$)-line bundle *L* on *X*. As remarked by Berman in [4], it is natural to generalise the notion of test configuration in terms of cohomology classes. In the polarised setting, the idea is to consider $(\mathcal {X}, c_1(\mathcal {L}))$ as a “test configuration” for $(X, c_1(L))$, by simply replacing $\mathcal {L}$ and *L* with their respective first Chern classes. This approach is motivated in detail below. Moreover, a number of basic and useful properties will be established, and throughout, this generalisation will systematically be compared to the original notion of algebraic test configuration $(\mathcal {X}, \mathcal {L})$ for (*X*, *L*), introduced by Donaldson in [27].

### Remark 3.1

Much of the following exposition goes through even when the cohomology class $\alpha$ is not Kähler. Unless explicitly stated otherwise, we thus assume that $\alpha = [\theta ]$ for some closed (1, 1)-form $\theta$ on *X*.

### Test Configurations for *X*

We first introduce the notion of test configuration $\mathcal {X}$ for *X*, working directly over $\mathbb {P}^1$. For the sake of comparison, recall the usual concept of test configuration for polarised manifolds, see, e.g. [12, 48]. In what follows, we refer to [31] for background on normal complex spaces.

#### Definition 3.2

A *test configuration* $\mathcal {X}$ *for* *X* consists of

- a normal *compact* Kähler complex space $\mathcal {X}$ with a flat morphism $\pi : \mathcal {X} \rightarrow \mathbb {P}^1$
- a $\mathbb {C}^*$-action $\lambda$ on $\mathcal {X}$ lifting the canonical action on $\mathbb {P}^1$
- a $$\mathbb {C}^*$$-equivariant isomorphism
  6 $$\begin{aligned} \mathcal {X} {\setminus } \mathcal {X}_0 \simeq X \times (\mathbb {P}^1 {\setminus } \{0\}). \end{aligned}$$

The isomorphism 6 gives an open embedding of $X \times (\mathbb {P}^1 {\setminus } \{0\})$ into $\mathcal {X}$, hence induces a canonical $\mathbb {C}^*$-equivariant bimeromorphic map $\mu : \mathcal {X} \dashrightarrow X \times \mathbb {P}^1$. We say that $\mathcal {X}$ dominates $X \times \mathbb {P}^1$ if the above bimeromorphic map $\mu$ is a morphism. Taking $\mathcal {X}'$ to be the normalisation of the graph of $\mathcal {X} \dashrightarrow X \times \mathbb {P}^1$ we obtain a $\mathbb {C}^*$-equivariant bimeromorphic morphism $\rho : \mathcal {X}' \rightarrow \mathcal {X}$ with $\mathcal {X}'$ normal and dominating $X \times \mathbb {P}^1$. In the terminology of [12] such a morphism $\rho$ is called a *determination* of $\mathcal {X}$. In particular, a determination of $\mathcal {X}$ always exists. By the above considerations we will often, up to replacing $\mathcal {X}$ by $\mathcal {X}'$, be able to assume that the given test configuration for *X* dominates $X \times \mathbb {P}^1$.

Moreover, any test configuration $\mathcal {X}$ for *X* can be dominated by a smooth test configuration $\mathcal {X}'$ for *X* (where we may even assume that $\mathcal {X}'_0$ is a divisor of simple normal crossings). Indeed, by Hironaka (see [33, Theorem 45] for the precise statement concerning normal complex spaces) there is a $\mathbb {C}^*$-equivariant proper bimeromorphic map $\mu : \mathcal {X}' \rightarrow \mathcal {X}$, with $\mathcal {X}'$ smooth, such that $\mathcal {X}_0'$ has simple normal crossings and $\mu$ is an isomorphism outside of the central fibre $\mathcal {X}_0$.

As a further consequence of the isomorphism (6), note that if $\Phi$ is a function on $\mathcal {X}$, then its restriction to each fibre $\mathcal {X}_{\tau } \simeq X$, $\tau \in \mathbb {P}^1 {\setminus } \{0\}$ identifies with a function on *X*. The function $\Phi$ thus gives rise to a family of functions $(\varphi _t)_{t \geqslant 0}$ on *X*, recalling our convention of reparametrising so that $t := - \log |\tau |$.

#### Remark 3.3

When *X* is projective (hence algebraic), the GAGA principle shows that the usual (i.e. algebraic, and normal) test configurations of *X* correspond precisely to the test configurations (in our sense of Definition 3.2) with $\mathcal {X}$ projective.

### Cohomological Test Configurations for $(X,\alpha )$

We now introduce a natural generalisation of the usual notion of *algebraic test configuration*$(\mathcal {X}, \mathcal {L})$ for a polarised manifold (*X*, *L*). This following definition involves the Bott–Chern cohomology on normal complex spaces, i.e. the space of locally $dd^c$-exact (1, 1)-forms (or currents) modulo globally $dd^c$-exact (1, 1)-forms (or currents). The Bott–Chern cohomology is finite dimensional and the cohomology classes can be pulled back. Moreover, $H^{1,1}_{\mathrm {BC}}(\mathcal {X}, \mathbb {R})$ coincides with the usual Dolbeault cohomology $H^{1,1}(\mathcal {X},\mathbb {R})$ whenever $\mathcal {X}$ is smooth. See, e.g. [11] for background.

#### Definition 3.4

A *cohomological test configuration for* $(X, \alpha )$ is a pair $(\mathcal {X},\mathcal {A})$ where $\mathcal {X}$ is a test configuration for *X* and $\mathcal {A} \in H^{1,1}_{\mathrm {BC}}(\mathcal {X},\mathbb {R})^{\mathbb {C}^*}$ is a $\mathbb {C}^*$-invariant (1, 1)-Bott–Chern cohomology class whose image under the $\mathbb {C}^*$-equivariant isomorphism

$$\begin{aligned} \mathcal {X} {\setminus } \mathcal {X}_0 \simeq X \times (\mathbb {P}^1 {\setminus } \{0\}). \end{aligned}$$

is $p_1^*\alpha$. Here $p_1: X \times \mathbb {P}^1 \rightarrow X$ is the first projection.

#### Definition 3.5

We say that a test configuration $(\mathcal {X}, \mathcal {A})$ for $(X,\alpha )$ is *smooth* if the total space $\mathcal {X}$ is smooth. In case $\alpha \in H^{1,1}(X,\mathbb {R})$ is Kähler, we say that $(\mathcal {X}, \mathcal {A})$ is *relatively Kähler* if the cohomology class $\mathcal {A}$ is relatively Kähler, i.e. there is a Kähler form $\beta$ on $\mathbb {P}^1$ such that $\mathcal {A} + \pi ^*\beta$ is Kähler on $\mathcal {X}$.

Exploiting the discussion following Definition 3.2 we *in practice* restrict attention to the situation when $(\mathcal {X}, \mathcal {A})$ is a smooth (cohomological) test configuration for $(X,\alpha )$ dominating $X \times \mathbb {P}^1$, with $\mu : \mathcal {X} \rightarrow X \times \mathbb {P}^1$ the corresponding $\mathbb {C}^*$-equivariant bimeromorphic morphism. This situation is studied in detail in Sect. 3.4, where we in particular show that the class $\mathcal {A} \in H^{1,1}(\mathcal {X}, \mathbb {R})$ is always of the form $\mathcal {A} = \mu ^*p_1^*\alpha + [D]$ for a unique $\mathbb {R}$-divisor *D* supported on the central fibre, cf. Proposition 3.10.

It is further natural to ask how the above notion of cohomological test configurations compares to the algebraic test configurations introduced by Donaldson in [27]. On the one hand, we have the following example:

#### Example 3.6

If $(\mathcal {Y}, \mathcal {L})$ is an algebraic test configuration for (*X*, *L*) and we let $\bar{\mathcal {Y}}$, $\bar{\mathcal {L}}$ and $\bar{L}$, respectively, denote the $\mathbb {C}^*$-equivariant compactifications over $\mathbb {P}^1$, then $(\bar{\mathcal {Y}}, c_1(\mathcal {L}))$ is a cohomological test configuration for $(X, c_1(L))$, canonically induced by $(\mathcal {Y}, \mathcal {L})$.

On the other hand, there is no converse such correspondence. For instance, even if (*X*, *L*) is a polarised manifold there are more cohomological test configurations $(\mathcal {X},\mathcal {A})$ for $(X,c_1(L))$ than algebraic test configurations $(\mathcal {Y}, \mathcal {L})$ for (*X*, *L*). However, we show in Proposition 3.14 that such considerations are not an issue in the study of K-semistability of $(X,\alpha )$.

### The Donaldson–Futaki Invariant and K-Semistability

The following generalisation of the Donaldson–Futaki invariant is straightforward, at least when the test configuration is smooth (in general one can use resolution of singularities to make sense of the intersection number below).

#### Definition 3.7

Let $(\mathcal {X}, \mathcal {A})$ be a cohomological test configuration for $(X,\alpha )$. The Donaldson–Futaki invariant of $(\mathcal {X}, \mathcal {A})$ is

$$\begin{aligned} \mathrm {DF}(\mathcal {X},\mathcal {A}) := \frac{\bar{\mathcal {S}}}{n+1}V^{-1} (\mathcal {A}^{n+1})_{\mathcal {X}} + V^{-1}(K_{\mathcal {X}/\mathbb {P}^1} \cdot \mathcal {A}^n)_{\mathcal {X}}. \end{aligned}$$

We recall that $\mathcal {X}$ is assumed to be *compact*, cf. Definition 3.2, and that $K_{\mathcal {X}/\mathbb {P}^1} := K_{\mathcal {X}} - \pi ^*K_{\mathbb {P}^1}$ denotes the relative canonical divisor. The point is that by results of Wang [53] and Odaka [39] $\mathrm {DF}(\bar{\mathcal {Y}},c_1(\mathcal {L}))$ coincides with $\mathrm {DF}(\mathcal {Y},\mathcal {L})$ whenever $(\mathcal {Y},\mathcal {L})$ is an algebraic test configuration for a polarised manifold (*X*, *L*), with $\mathcal {Y}$ normal (see the proof of Proposition 3.14). Hence the above quantity is a generalisation of the classical Donaldson–Futaki invariant.

The analogue of K-semistability in the context of cohomological test configurations is defined as follows.

#### Definition 3.8

We say that $(X,\alpha )$ is * K-semistable* if $\mathrm {DF}(\mathcal {X},\mathcal {A}) \geqslant 0$ for all relatively Kähler test configurations $(\mathcal {X},\mathcal {A})$ for $(X,\alpha )$.

#### Remark 3.9

With the study of K-semistability in mind, we emphasise that the Donaldson–Futaki invariant $\mathrm {DF}(\mathcal {Y},\mathcal {L})$ (cf. [39, 53]) depends only on $\mathcal {Y}$ and $c_1(\mathcal {L})$. The notion of cohomological test configuration emphasises this fact.

In order to further motivate the above definitions, we now introduce a number of related concepts and basic properties that will be useful in the sequel.

### Test Configurations Characterised by $\mathbb {R}$-Divisors

Recall that if $(\mathcal {X},\mathcal {L})$ is an algebraic test configuration for a polarised manifold (*X*, *L*) that dominates $(X,L) \times \mathbb {C}$, then $\mathcal {L} = \mu ^* p_1^*L + D$ for a unique $\mathbb {Q}$-Cartier divisor *D* supported on $\mathcal {X}_0$, see [12]. Similarly, the following result characterises the classes $\mathcal {A}$ associated with smooth and dominating cohomological test configurations, in terms of $\mathbb {R}$-divisors *D* supported on the central fibre $\mathcal {X}_0$.

#### Proposition 3.10

Let $(\mathcal {X}, \mathcal {A})$ be a smooth cohomological test configuration for $(X, \alpha )$ dominating $X \times \mathbb {P}^1$, with $\mu : \mathcal {X} \rightarrow X \times \mathbb {P}^1$ the corresponding canonical $\mathbb {C}^*$-equivariant bimeromorphic morphism. Then there exists a unique $\mathbb {R}$-divisor *D* supported on the central fibre $\mathcal {X}_0$ such that

$$\begin{aligned} \mathcal {A} = \mu ^*p_1^*\alpha + [D] \end{aligned}$$

in $$H^{1,1}(\mathcal {X}, \mathbb {R})$$.

#### Proof

Let $\alpha := [\omega ] \in H^{1,1}(X,\mathbb {R})$. We begin by proving existence: By hypothesis $\mathcal {X}$ dominates $X \times \mathbb {P}^1$ via the morphism $\mu$, such that the central fibre decomposes into the strict transform of $X \times \{0\}$ and the $\mu$-exceptional divisor. We write $\mathcal {X}_0 = \sum _i b_i E_i$, with $E_i$ irreducible. Denoting by [*E*] the cohomology class of *E* and by $p_1: X \times \mathbb {P}^1 \rightarrow X$ the projection on the first factor, we then have the following formula: $\square$

#### Lemma 3.11

$$H^{1,1}(\mathcal {X}) = \mu ^*p_1^*H^{1,1}(X) \; \oplus \; \bigoplus _i \mathbb {R} [E_i]$$.

#### Proof of Lemma 3.11

Let $\Theta$ be a closed (1, 1)-form on $\mathcal {X}$. Then $T := \Theta - \mu ^*(\mu _*\Theta )$ is a closed (1, 1)-current of order 0 supported on $\cup _i E_i = \mathrm {Exc}(\mu )$. By Demailly’s second theorem of support (see [23]) it follows that $T = \sum _i \lambda _i \delta _{E_j}$ and hence $[\Theta ] = \mu ^*(\mu _*[\Theta ]) + \sum _i \lambda _i [E_i]$ in $H^{1,1}(\mathcal {X})$.

Since $H^{1,1}(\mathbb {P}^1)$ is generated by [0], we have $p_2^*H^{1,1}(\mathbb {P}^1) = \mathbb {R}[X \times \{0\}]$. By the Künneth formula, it thus follows that $H^{1,1}(\mathcal {X}) = \mu ^*p_1^*H^{1,1}(X) \; \oplus \; \mu ^*(\mathbb {R}[X \times \{0\}])\; \oplus \; \bigoplus _i \mathbb {R} [E_i]$. $\square$

If we decompose $\mathcal {A}$ accordingly we obtain $\mathcal {A} = \mu ^*p_1^*\eta + [D]$ with $D := \mu ^*(c[X \times \{0\}]) + \bigoplus _i b_i [E_i]$ and $\eta$ a class in $H^{1,1}(X)$. The restrictions of $\mathcal {A}$ and $\mu ^*p_1^*\alpha$ to $\pi ^{-1}(1) \simeq X \times \{1\} \simeq X$ are identified with $\alpha$ and $\eta$, respectively. Since *D* is supported on $\mathcal {X}_0$ it follows that $\eta = \alpha$. We thus have the sought decomposition, proving existence.

As for the uniqueness, we let $\mathcal {D}_0$ be the set of $\mathbb {R}$-divisors *D* with support contained in the central fibre $\mathcal {X}_0$. Consider the linear map

$$\begin{aligned} R: \mathcal {D}_0\rightarrow & {} H^{1,1}(\mathcal {X})\\ D\mapsto & {} [D] \end{aligned}$$

The desired uniqueness property is equivalent to injectivity of *R*. Hence, assume that $[D] = 0$ in $H^{1,1}(\mathcal {X})$. In particular $D_{\vert E_i} \equiv 0$ and it follows from a corollary of Zariski’s lemma (see, e.g. [1, Lemma 8.2]) that $D = c\mathcal {X}_0$, with $c \in \mathbb {R}$. But, letting $\beta$ be any Kähler form on *X*, we see from the projection formula that

$$\begin{aligned} (\mathcal {X}_0 \cdot (\mu ^*p_1^*\beta )^n)_{\mathcal {X}} = ((X \times \{0\}) \cdot (p_1^*\beta )^n)_{X \times \mathbb {P}^1} = \beta ^n = V > 0, \end{aligned}$$

since *V* is the Kähler volume. Hence $[\mathcal {X}_0]$ is a non-zero class in $H^{1,1}(\mathcal {X})$. It follows that $c = 0$, thus $D = 0$ as well. We are done. $\square$

This gives a very convenient characterisation of smooth cohomological test configurations for $(X,\alpha )$ that dominate $X \times \mathbb {P}^1$.

In what follows, we will make use of resolution of singularities to associate a new test configuration $(\mathcal {X}', \mathcal {A}')$ for $(X,\alpha )$ to a given one, noting that this can be done *without changing the Donaldson–Futaki invariant*. Indeed, by Hironaka [33, Theorem 45] (see also Sect. 3.2) there is a $\mathbb {C}^*$-equivariant proper bimeromorphic map $\mu : \mathcal {X}' \rightarrow \mathcal {X}$, with $\mathcal {X}'$ smooth and such that $\mathcal {X}_0'$ has simple normal crossings. Moreover, $\mu$ is an isomorphism outside of the central fibre $\mathcal {X}_0$. Set $\mathcal {A}' := \mu ^*\mathcal {A}$. By the projection formula we then have

$$\begin{aligned} \mathrm {DF}(\mathcal {X}', \mathcal {A}')= & {} \frac{\bar{\mathcal {S}}}{n+1}V^{-1} ((\mathcal {A}')^{n+1})_{\mathcal {X}'} + V^{-1}(K_{\mathcal {X}'/\mathbb {P}^1} \cdot (\mathcal {A}')^n)_{\mathcal {X}'}\\= & {} \frac{\bar{\mathcal {S}}}{n+1}V^{-1}(\mathcal {A}^{n+1})_{\mathcal {X}} + V^{-1}(K_{\mathcal {X}/\mathbb {P}^1} \cdot \mathcal {A}^n)_{\mathcal {X}} = \mathrm {DF}(\mathcal {X}, \mathcal {A}). \end{aligned}$$

The following result states that it suffices to test K-semistability for a certain class of cohomological test configurations ‘characterised by an $\mathbb {R}$-divisor’ (in the above sense of Proposition 3.10).

#### Proposition 3.12

Let $\alpha \in H^{1,1}(X,\mathbb {R})$ be Kähler. Then $(X,\alpha )$ is K-semistable if and only if $\mathrm {DF}(\mathcal {X},\mathcal {A}) \geqslant 0$ for all smooth, relatively Kähler cohomological test configurations $(\mathcal {X},\mathcal {A})$ for $(X, \alpha )$ dominating $X \times \mathbb {P}^1$.

#### Proof

Let $(\mathcal {X},\mathcal {A})$ be any cohomological test configuration for $(X, \alpha )$ that is relatively Kähler. By Hironaka (see [33]) there is a sequence of blowups $\rho : \mathcal {X}' \rightarrow X \times \mathbb {P}^1$ with smooth $\mathbb {C}^*$-equivariant centres such that $\mathcal {X}'$ simultaneously dominates $\mathcal {X}$ and $X \times \mathbb {P}^1$ via morphisms $\mu$ and $\rho$, respectively. Moreover, there is a divisor *E* on $\mathcal {X}'$ that is $\rho$-exceptional and $\rho$-ample (and antieffective, i.e. $-E$ is effective). By Proposition 3.10, we have

$$\begin{aligned} \mu ^*\mathcal {A} = \rho ^*p_1^*\alpha + [D], \end{aligned}$$

where *D* is an $\mathbb {R}$-divisor on $\mathcal {X}'$ supported on $\mathcal {X}'_0$. Note that the class $\mu ^*\mathcal {A} \in H^{1,1}(\mathcal {X}', \mathbb {R})$ is relatively nef.

We proceed by perturbation. Since $\alpha$ is Kähler on *X*, we may pick a Kähler class $\eta$ on $\mathbb {P}^1$ such that $p_1^*\alpha + p_2^*\eta =: \beta$ is Kähler on $X \times \mathbb {P}^1$. Since *E* is $\rho$-ample one may in turn fix an $\varepsilon \in (0,1)$ sufficiently small such that $\rho ^*\beta + \varepsilon [E]$ is Kähler on $\mathcal {X}'$. It follows that $\rho ^*p_1^*\alpha + \varepsilon [E]$ is *relatively* Kähler (with respect to $\mathbb {P}^1$) on $\mathcal {X}'$. Thus $\rho ^*p_1^*\alpha + [D] + \delta (\rho ^*p_1^*\alpha + \varepsilon [E])$ is relatively Kähler for all $\delta \geqslant 0$ small enough. In turn, so is $\mathcal {A}'_{\delta } := \rho ^*p_1^*\alpha + [D_{\delta }]$, where $D_{\delta }$ denotes the convex combination $D_{\delta } := \frac{1}{1+\delta }D + \frac{\delta \varepsilon }{1 + \delta }E$. Assuming that the $\mathrm {DF}$-invariant of a smooth and dominating test configuration is always non-negative, it follows from the projection formula and continuity of the Donaldson–Futaki invariant, that

$$\begin{aligned} 0 \leqslant \mathrm {DF}(\mathcal {X}',\mathcal {A}'_{\delta }) \longrightarrow \mathrm {DF}(\mathcal {X}',\mu ^*\mathcal {A}) = \mathrm {DF}(\mathcal {X},\mathcal {A}) \end{aligned}$$

as $\delta \rightarrow 0$. The other direction holds by definition, so this proves the first part of the lemma. $\square$

#### Remark 3.13

With respect to testing K-semistability one can in fact restrict the class of test configurations that need to be considered even further, as explained in Sect. 3.6.

### Cohomological K-Semistability for Polarised Manifolds

It is useful to compare cohomological and algebraic K-semistability in the special case of a polarised manifold (*X*, *L*).

#### Proposition 3.14

Let (*X*, *L*) be a polarised manifold and let $\alpha := c_1(L)$. Then $(X,c_1(L))$ is (*cohomologically*) K-semistable if and only if (*X*, *L*) is (*algebraically*) K-semistable.

#### Proof

Suppose that $(X, c_1(L))$ is cohomologically K-semistable. If $(\mathcal {X}, \mathcal {L})$ is an ample test configuration for (*X*, *L*), let $\mathcal {A} := c_1(\bar{\mathcal {L}})$. By the intersection theoretic characterisation of the Donaldson–Futaki invariant (Definition 3.7) we then have $\mathrm {DF}(\mathcal {X},\mathcal {A}) = \mathrm {DF}(\mathcal {X},\mathcal {L}) \geqslant 0$. Hence (*X*, *L*) is algebraically K-semistable.

Conversely, suppose that (*X*, *L*) is algebraically K-semistable and let $(\mathcal {X}, \mathcal {A})$ be a cohomological test configuration for $(X,\alpha )$. By Lemma 3.12 we may assume that $(\mathcal {X}, \mathcal {A})$ is a smooth, relatively Kähler test configuration for $(X,\alpha )$ dominating $X \times \mathbb {P}^1$, with $\mu : \rightarrow X \times \mathbb {P}^1$ the corresponding $\mathbb {C}^*$-equivariant bimeromorphic morphism. By Proposition 3.10 we further have $\mathcal {A} = \mu ^*p_1^*c_1(L) + [D]$ for a uniquely determined $\mathbb {R}$-divisor *D* on $\mathcal {X}$ supported on the central fibre $\mathcal {X}_0$. Since $\mathcal {A}$ is relatively Kähler, there is a Kähler form $\eta$ on $\mathbb {P}^1$ such that $\mathcal {A} + \pi ^*\eta$ is Kähler on $\mathcal {X}$. Approximating the coefficients of the divisor *D* by a sequence of rationals, we write $D = \lim D_j$ for $\mathbb {Q}$-divisors $D_j$ on $\mathcal {X}$, all supported on $\mathcal {X}_0$. As $j \rightarrow +\infty$, we then have

$$\begin{aligned} \mu ^*p_1^*c_1(L) + [D_j] + \pi ^*\eta \longrightarrow \mathcal {A} + \pi ^*\eta , \end{aligned}$$

which is a Kähler form on $\mathcal {X}$. Since the Kähler cone is open, it follows that $\mu ^*p_1^*c_1(L) + [D_j] + \pi ^*\eta$ is also Kähler for all *j* large enough.

Now let $\mathcal {L}_j := \mu ^*p_1^*L + D_j$. By the above, $\mathcal {L}_j$ is a relatively ample $\mathbb {Q}$-line bundle over $\mathcal {X}$ and $c_1(\mathcal {L}_j) \rightarrow \mathcal {A}$. We thus conclude that $(\mathcal {X}, \mathcal {L}_j)$ (for all *j* large enough) is an ample test configuration for (*X*, *L*). Hence

$$\begin{aligned} 0 \leqslant \mathrm {DF}(\mathcal {X}, \mathcal {L}_j) \longrightarrow \mathrm {DF}(\mathcal {X}, \mathcal {A}) \end{aligned}$$

as $j \rightarrow +\infty$, which is what we wanted to prove. $\square$

### The Non-Archimedean Mabuchi Functional and Base Change

Let $(\mathcal {X},\mathcal {A})$ be a cohomological test configuration for $(X,\alpha )$. A natural operation on $(\mathcal {X}, \mathcal {A})$ is that of *base change* (on $\mathcal {X}$ and we pull back $\mathcal {A}$). Unlike resolution of singularities, however, the $\mathrm {DF}$-invariant does not behave well under base change. In this context, a more natural object of study is instead the *non-Archimedean Mabuchi functional*$\mathrm {M}^{\mathrm {NA}}$ (first introduced in [12, 13], where also an explanation of the terminology is given).

#### Definition 3.15

The *non-Archimedean Mabuchi functional* is the modification of the Donaldson–Futaki invariant given by

$$\begin{aligned} \mathrm {M}^{\mathrm {NA}}(\mathcal {X}, \mathcal {A}) := \mathrm {DF}(\mathcal {X}, \mathcal {A}) + V^{-1}((\mathcal {X}_{0,\mathrm {red}} - \mathcal {X}_0) \cdot \mathcal {A}^n)_{\mathcal {X}}. \end{aligned}$$

Note that the ‘correction term’ $V^{-1}((\mathcal {X}_{0,\mathrm {red}} - \mathcal {X}_0) \cdot \mathcal {A}^n)_{\mathcal {X}}$ is non-positive and vanishes precisely when the central fibre $\mathcal {X}_0$ is reduced. The point of adding to $\mathrm {DF}$ this additional term is that the resulting quantity $\mathrm {M}^{\mathrm {NA}}(\mathcal {X}, \mathcal {A})$ becomes *homogeneous under base change*, i.e. we have the following lemma.

#### Lemma 3.16

([12]) Let $(\mathcal {X},\mathcal {A})$ be a cohomological test configuration for $(X,\alpha )$ and let $d \in \mathbb {N}$. Denote by $\mathcal {X}_d$ the normalisation of the base change of $\mathcal {X}$, by $g_d: \mathcal {X}_d \rightarrow \mathcal {X}$ the corresponding morphism (of degree *d*) and set $\mathcal {A}_d := g_d^*\mathcal {A}$. Then

$$\begin{aligned} \mathrm {M}^{\mathrm {NA}}(\mathcal {X}_d, \mathcal {A}_d) = d \cdot \mathrm {M}^{\mathrm {NA}}(\mathcal {X}, \mathcal {A}). \end{aligned}$$

#### Proof

We refer the reader to [12, Proposition 7.13], whose proof goes through in the analytic case as well. $\square$

As an application, it follows from Mumford’s semistable reduction theorem ([32, p. 53], see also [34, §16, p. 6] for a remark on the analytic case) that there is a $d \in \mathbb {N}$, a finite base change $f:\tau \mapsto \tau ^d$ (for *d* ‘divisible enough’), a smooth test configuration $\mathcal {X}'$ and a diagram 
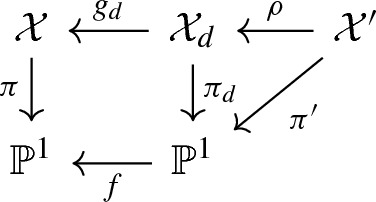
 such that $\mathcal {X}'$ is semistable, i.e. smooth and such that $\mathcal {X}_0'$ is a reduced divisor with simple normal crossings. In particular, note that the correction term $V^{-1}((\mathcal {X}_{0,\mathrm {red}}' - \mathcal {X}_0') \cdot \mathcal {A}^n)_{\mathcal {X}'}$ vanishes. Here $\mathcal {X}_d$ denotes the normalisation of the base change, which is dominated by the semistable test configuration $\mathcal {X}'$ for *X*. Moreover, $g_d \circ \rho$ is an isomorphism over $\mathbb {P}^1 {\setminus } \{0\}$.

Letting $\mathcal {A}_d := g_d^*\mathcal {A}$ be the pullback of $\mathcal {A}$ to $\mathcal {X}_d$, and $\mathcal {A}' := \rho ^* \mathcal {A}_d$ the pullback to $\mathcal {X}'$, it follows from the above homogeneity of the $\mathrm {M}^{\mathrm {NA}}$ that

$$\begin{aligned} \mathrm {DF}(\mathcal {X}', \mathcal {A}') = \mathrm {M}^{\mathrm {NA}}(\mathcal {X}', \mathcal {A}') = \mathrm {M}^{\mathrm {NA}}(\mathcal {X}_d, \mathcal {A}_d) = d \cdot \mathrm {M}^{\mathrm {NA}}(\mathcal {X}, \mathcal {A}) \leqslant d \cdot \mathrm {DF}(\mathcal {X}, \mathcal {A}), \end{aligned}$$

where *d* is the degree of $g_d$. We have thus associated with $(\mathcal {X}, \mathcal {A})$ a new test configuration $(\mathcal {X}', \mathcal {A}')$ for $(X,\alpha )$ such that the total space $\mathcal {X}'$ is semistable. Up to replacing $\mathcal {X}'$ with a *determination* (see Sect. 3.1) we can moreover assume that $\mathcal {X}'$ dominates $X \times \mathbb {P}^1$. Hence, the above shows that $\mathrm {DF}(\mathcal {X}, \mathcal {A}) \geqslant \mathrm {DF}(\mathcal {X}', \mathcal {A}')/d$. By an argument by perturbation much as the one in the proof of Proposition 3.12, we obtain the following stronger version of the aforementioned result.

#### Proposition 3.17

Let $\alpha \in H^{1,1}(X,\mathbb {R})$ be Kähler. Then $(X,\alpha )$ is K-semistable (Definition 3.8) if and only if $\mathrm {DF}(\mathcal {X}, \mathcal {A}) \geqslant 0$ for all semistable, relatively Kähler cohomological test configurations $(\mathcal {X}, \mathcal {A})$ for $(X, \alpha )$ dominating $X \times \mathbb {P}^1$.

## Transcendental Kempf–Ness Type Formulas

Let *X* be a compact Kähler manifold of dimension *n* and let $\theta _i$, $0 \leqslant i \leqslant n$, be closed (1, 1)-forms on *X*. Let $\alpha _i := [\theta _i] \in H^{1,1}(X,\mathbb {R})$ be the corresponding cohomology classes. In this section we aim to prove Theorem B. In other words, we establish a Kempf–Ness type formula (for *cohomological* test configurations), which connects the asymptotic slope of the multivariate energy functional $\langle \varphi _0^t,\dots ,\varphi _n^t \rangle _{(\theta _0, \dots , \theta _n)}$ (see Definition 2.2) with a certain intersection number. In order for such a result to hold, we need to ask that the rays $(\varphi _i^t)_{t \geqslant 0}$ are *compatible* with $(\mathcal {X}_i, \mathcal {A}_i)$ in a sense that has to do with extension across the central fibre, see Sect. 4.1.

For what follows, note that, by equivariant resolution of singularities, there is a test configuration $\mathcal {X}$ for *X* which is smooth and dominates $X \times \mathbb {P}^1$. This setup comes with *canonical*$\mathbb {C}^*$-equivariant bimeromorphic maps $\rho _i: \mathcal {X} \rightarrow \mathcal {X}_i$, respectively. In particular:

### Definition 4.1

We define the intersection number

$$\begin{aligned} (\mathcal {A}_0 \cdot \dots \cdot \mathcal {A}_n) := (\rho _0^* \mathcal {A}_0 \cdot \dots \cdot \rho _n^* \mathcal {A}_n)_{\mathcal {X}} \end{aligned}$$

by means of pulling back the respective cohomology classes to $\mathcal {X}$.

### Remark 4.2

Up to desingularising we can and we will in this section consider only smooth cohomological test configurations $(\mathcal {X}_i,\mathcal {A}_i)$ for $(X, \alpha _i)$ dominating $X \times \mathbb {P}^1$, with $\mu _i: \mathcal {X}_i \rightarrow X \times \mathbb {P}^1$ the corresponding $\mathbb {C}^*$-equivariant bimeromorphic morphisms, respectively. We content ourselves by noting that the following $\mathcal {C}^{\infty }$-compatibility condition can be defined (much in the same way, using a desingularisation) in the singular case as well.

### Compatibility of Rays and Test Configurations

Let $(\mathcal {X},\mathcal {A})$ be a smooth (cohomological) test configuration for $(X,\alpha )$ dominating $X \times \mathbb {P}^1$, with $\mu :\mathcal {X} \rightarrow X \times \mathbb {P}^1$ the corresponding canonical $\mathbb {C}^*$-equivariant bimeromorphic morphism. We then have

$$\begin{aligned} \mathcal {A}=\mu ^*p_1^*\alpha +[D] \end{aligned}$$

for a unique $\mathbb {R}$-divisor *D* supported on $\mathcal {X}_0$, with $p_1:X \times \mathbb {P}^1 \rightarrow X$ denoting the first projection, cf. Proposition 3.10.

We fix the choice of an $S^1$-invariant function ‘Green function’ $\psi _D$ for *D*, so that $\delta _D=\theta _D+dd^c\psi _D$, with $\theta _D$ a smooth $S^1$-invariant closed (1, 1)-form on $\mathcal {X}$. Locally, we thus have

$$\begin{aligned} \psi _D = \sum _j a_j \log |f_j| \; \; \text {mod} \; \mathcal {C}^{\infty }, \end{aligned}$$

where (writing $D := \sum _j a_jD_j$ for the decomposition of *D* into irreducible components) the $f_j$ are local defining equations for the $D_j$, respectively. In particular, the choice of $\psi _D$ is a uniquely determined modulo smooth function.

The main purpose of this section is to establish Theorem B, which is a formula relating algebraic (intersection theoretic) quantities to asymptotic slopes of Deligne functionals (e.g. $\mathrm {E}$ or $\mathrm {J}$) along certain rays. However, such a formula cannot hold for *any* such ray. The point of the following *compatibility conditions* is to establish some natural situations in which this formula holds. Technically, recall that a ray $(\varphi _t)_{t \geqslant 0}$ on *X* is in correspondence with an $S^1$-invariant functions $\Phi$ on $X \times \bar{\Delta }^*$. The proof of Theorem B, will show that it is important to extend the function $\Phi \circ \mu$ on $\mathcal {X} {\setminus } \mathcal {X}_0$ also across the central fibre $\mathcal {X}_0$.

To this end, we introduce the notions of $\mathcal {C}^{\infty }$-, $L^{\infty }$- and $\mathcal {C}^{1,\bar{1}}$-compatibility between the ray $(\varphi _t)_{t \geqslant 0}$ and the test configuration $(\mathcal {X}, \mathcal {A})$. The purpose of introducing more than one version of compatibility is that we will distinguish between the following two situations of interest to us:

(i) smooth but not necessarily subgeodesic rays $(\varphi _t)$ that are $\mathcal {C}^{\infty }$-compatible with the smooth test configuration $(\mathcal {X}, \mathcal {A})$ for $(X,\alpha )$, dominating $X \times \mathbb {P}^1$. Here we can consider $\alpha = [\theta ] \in H^{1,1}(X,\mathbb {R})$ for any closed (1, 1)-form $\theta$ on *X*.
(ii) locally bounded *subgeodesic* rays $(\varphi _t)$ that are $L^{\infty }$-compatible or (more restrictively) $\mathcal {C}^{1,\bar{1}}$-compatible with the given smooth and *relatively Kähler* test configuration $(\mathcal {X}, \mathcal {A})$ for $(X,\alpha )$, dominating $X \times \mathbb {P}^1$. Here we thus suppose that $\alpha$ is a Kähler class.

Theorem B has valid formulations in both these situations, as pointed out in Remark 4.11. The second situation is interesting notably with weak geodesic rays in mind, cf. Sect. 4.3.

### $\mathcal {C}^{\infty }$-Compatible Rays

We first introduce the notion of smooth (not necessarily subgeodesic) rays that are $\mathcal {C}^{\infty }$*-compatible* with the given test configuration $(\mathcal {X}, \mathcal {A})$ for $(X,\alpha )$.

#### Definition 4.3

Let $(\varphi _t)_{t \geqslant 0}$ be a smooth ray in $\mathcal {C}^{\infty }(X)$ and denote by $\Phi$ the corresponding smooth $S^1$-invariant function on $X \times \bar{\Delta }^*$. We say that $(\varphi _t)$ and $(\mathcal {X},\mathcal {A})$ are $\mathcal {C}^{\infty }$*-compatible* if $\Phi \circ \mu +\psi _D$ extends smoothly across $\mathcal {X}_0$.

The condition is indeed independent of the choice of $\psi _D$, as the latter is a well-defined modulo smooth function. In the case of a polarised manifold (*X*, *L*) with an (algebraic) test configuration $(\mathcal {X}, \mathcal {L})$ this condition amounts to demanding that the metric on $\mathcal {L}$ associated with the ray $(\varphi _t)_{t \geqslant 0}$ extends smoothly across the central fibre.

As a useful ‘model example’ to keep in mind, let $\Omega$ be a smooth $S^1$-invariant representative of $\mathcal {A}$ and denote the restrictions $\Omega _{\vert \mathcal {X}_{\tau }} =: \Omega _{\tau }$. Note that $\Omega _{\tau }$ and $\Omega _1$ are cohomologous for each $\tau \in \mathbb {P}^1 {\setminus } \{0\}$, and hence we may define a ray $(\varphi _t)_{t \geqslant 0}$ on *X*, $\mathcal {C}^{\infty }$-compatible with $(\mathcal {X}, \mathcal {A})$, by the following relation $\lambda (\tau )^*\Omega _{\tau } - \Omega _1 = dd^c\varphi _{\tau }$, where $t = -\log |\tau |$ and $\lambda (\tau ): \mathcal {X}_{\tau } \rightarrow \mathcal {X}_1 \simeq X$ is the isomorphism induced by the $\mathbb {C}^*$-action $\lambda$ on $\mathcal {X}$.

We further establish existence of a smooth $\mathcal {C}^{\infty }$-compatible *subgeodesic* ray associated to a given relatively Kähler test configuration $(\mathcal {X}, \mathcal {A})$ for $(X,\alpha )$.

#### Lemma 4.4

If $\mathcal {A}$ is relatively Kähler, then $(\mathcal {X},\mathcal {A})$ is $\mathcal {C}^{\infty }$-compatible with some smooth subgeodesic ray $(\varphi _t)$.

#### Proof

Since $\mathcal {A}$ is relatively Kähler, it admits a smooth $S^1$-invariant representative $\Omega$ with $\Omega +\pi ^*\eta >0$ for some $S^1$-invariant Kähler form $\eta$ on $\mathbb {P}^1$. By the $dd^c$-lemma on $\mathcal {X}$, we have $\Omega =\mu ^*p_1^*\omega +\theta _D+dd^c u$ for some $S^1$-invariant $u\in \mathcal {C}^{\infty }(X)$, which may be assumed to be 0 after replacing $\psi _D$ with $\psi _D-u$. As a result, we get

$$\begin{aligned} \Omega =\mu ^*p_1^*\omega +\delta _D-dd^c\psi _D. \end{aligned}$$

We may also choose a smooth $S^1$-invariant function *f* on a neighbourhood *U* of $\bar{\Delta }$ such that $\eta _{\vert U}=dd^c f$, and a constant $A \gg 1$ such that $D\leqslant A\mathcal {X}_0$. Using the Lelong–Poincaré formula $\delta _{\mathcal {X}_0}=dd^c\log |\tau |$ we get

$$\begin{aligned} 0<\Omega +\pi ^*\eta =\mu ^*p_1^*\omega +\delta _{D-A\mathcal {X}_0}+dd^c\left( f\circ \pi +A\log |\tau |-\psi _D\right) \end{aligned}$$

on $\pi ^{-1}(U)$. Since $D-A\mathcal {X}_0\leqslant 0$, it follows that $f\circ \pi +A\log |\tau |-\psi _D$ is $\mu ^*p_1^*\omega$-psh, and hence descends to an $S^1$-invariant $p_1^*\omega$-psh function $\tilde{\Phi }$ on $X\times U$ (because the fibres of $\mu$ are compact and connected, by Zariski’s main theorem). The ray associated with the $S^1$-invariant function $\Phi :=\tilde{\Phi }-A\log |\tau |$ has the desired properties. $\square$

### $\mathcal {C}^{1,\bar{1}}$-Compatible Rays and the Weak Geodesic Ray Associated with $(\mathcal {X}, \mathcal {A})$

Let $(\mathcal {X}, \mathcal {A})$ be a smooth, relatively Kähler cohomological test configuration for $(X,\alpha )$ (with $\alpha$ Kähler). With this setup, it is also interesting to consider the following weaker compatibility conditions, referred to as $L^{\infty }$-compatibility and $\mathcal {C}^{1,\bar{1}}$-compatibility, respectively.

#### Definition 4.5

Let $(\varphi _t)_{t \geqslant 0}$ be a locally bounded subgeodesic ray, and denote by $\Phi$ the corresponding $S^1$-invariant locally bounded $p_1^*\omega$-psh function on $X\times \bar{\Delta }^*$. We say that $(\varphi _t)$ and $(\mathcal {X},\mathcal {A})$ are $L^{\infty }$*-compatible* if $\Phi \circ \mu +\psi _D$ is locally bounded near $\mathcal {X}_0$, resp. $\mathcal {C}^{1,\bar{1}}$*-compatible* if $\Phi \circ \mu +\psi _D$ is of class $\mathcal {C}^{1,\bar{1}}$ on $\pi ^{-1}(\Delta )$.

Indeed, we will see that $\mathcal {C}^{1,\bar{1}}$-compatibility is always satisfied for weak geodesic rays associated with $(\mathcal {X}, \mathcal {A})$. In particular, for any given test configuration, $\mathcal {C}^{1,\bar{1}}$-compatible subgeodesics always exist. This is the content of the following result, which is a consequence of the theory for degenerate Monge–Ampère equations on manifolds with boundary. We refer the reader to [10] for the relevant background.

#### Lemma 4.6

With the situation (2) in mind, let $(\mathcal {X},\mathcal {A})$ be a smooth, relatively Kähler cohomological test configuration of $(X,\alpha )$ dominating $X \times \mathbb {P}^1$. Then $(\mathcal {X}, \mathcal {A})$ is $\mathcal {C}^{1,\bar{1}}$-compatible with some weak geodesic ray $(\varphi _t)_{t \geqslant 0}$.

#### Remark 4.7

The proof will show that the constructed ray is actually unique, once a $\varphi _0 \in \mathcal {H}$ is fixed.

#### Proof of Lemma 4.6

Let $M := \pi ^{-1}(\bar{\Delta }) \subset \mathcal {X}$. It is a smooth complex manifold with boundary $\partial M = \pi ^{-1}(S^1)$.

Let *D*, $\theta _D$, $\psi _D$ and $\Omega$ be as above. Since $\Omega$ is relatively Kähler there is an $\eta \in H^{1,1}(\mathbb {P}^1)$ such that $\Omega + \pi ^*\eta$ is Kähler on $\mathcal {X}$. We may then write $\tilde{\Omega } = \Omega + \pi ^*\eta + dd^cg$, where $\tilde{\Omega }$ is a Kähler form on $\mathcal {X}$ and $g \in \mathcal {C}^{\infty }(\mathcal {X})$. In a neighbourhood of $\bar{\Delta }$ the form $\eta$ is further $dd^c$-exact, and so we write $\eta = dd^c (g' \circ \pi )$ for a smooth function $g' \circ \pi$ on $\bar{\Delta }$. We now consider the following degenerate complex Monge–Ampère equation:

$$\begin{aligned} (\star ) \left\{ \begin{array}{ll} (\tilde{\Omega } + dd^c\tilde{\Psi })^{n+1} = 0 \; \; \mathrm {on}\; \mathrm {Int}(M) \\ \tilde{\Psi }_{\vert \partial M} = \varphi _0 + \psi _D - g' -g \end{array}. \right. \end{aligned}$$

Since $\tilde{\Omega }$ is Kähler, it follows that there exists a unique $\tilde{\Omega }$-psh function $\tilde{\Psi }$ solving $(\star )$ and that is moreover of class $\mathcal {C}^{1,\bar{1}}$ (see for instance [10, Theorem B]. We now define a $p_1^*\omega$-psh function on $X \times \bar{\Delta }^* \hookrightarrow \mathcal {X}$ by $\mu ^*\Phi = \tilde{\Psi } - \psi _D + g' + g$. We then have

$$\begin{aligned} \mu ^*(p_1^*\omega + dd^c\Phi ) = \tilde{\Omega } + dd^c\tilde{\Psi } \end{aligned}$$

on $\pi ^{-1}(\bar{\Delta }^*)$. In particular, $\Phi$ defines a weak geodesic ray $(\varphi _t)_{t \geqslant 0}$ on *X*. Moreover, the current

$$\begin{aligned} \mu ^*dd^c\Phi + \delta _D = dd^c\tilde{\Psi } + \delta _D - dd^c\psi _D = dd^c\tilde{\Psi } + \theta _D \end{aligned}$$

has locally bounded coefficients. Indeed, $dd^c\tilde{\Psi } \in L^{\infty }_{\mathrm {loc}}$ (as solution of $(\star )$, cf. [10]) and $\theta _D$ is a smooth (1, 1)-form on $\bar{\mathcal {X}}$. The constructed ray is thus $\mathcal {C}^{1,\bar{1}}$-compatible with $(\mathcal {X}, \mathcal {A})$. $\square$

### A Useful Lemma

We now note that in order to compute the asymptotic slope of the Monge–Ampère energy functional $$\mathrm {E}$$ or its multivariate analogue $$\mathrm {E}_{(\omega _0, \dots , \omega _n)}$$ we may in fact replace $$L^{\infty }$$-compatible rays $$(\varphi ^t)$$ with $$(\mathcal {X}, \mathcal {A})$$ by $$\mathcal {C}^{\infty }$$-compatible ones. Indeed, note that any two locally bounded subgeodesic rays $$(\varphi _t)$$ and $$(\varphi '_t)$$$$L^{\infty }$$-compatible with $$(\mathcal {X},\mathcal {A})$$ satisfy $$\Phi \circ \mu =\Phi '\circ \mu +O(1)$$ near $$\mathcal {X}_0$$, and hence $$\varphi _t=\varphi '_t+O(1)$$ as $$t\rightarrow +\infty $$. This leads to the following observation, which will be useful in the view of proving Theorems B and C.

#### Lemma 4.8

Let $(\mathcal {X}_i, \mathcal {A}_i)$ be smooth, relatively Kähler cohomological test configurations for $(X,\alpha _i)$, respectively, dominating $X \times \mathbb {P}^1$. Let $(\varphi _i^t)_{t \geqslant 0}$ and $({\varphi '}_i^t)_{t \geqslant 0}$ be locally bounded subgeodesics that are $L^{\infty }$-compatible with $(\mathcal {X}_i, \mathcal {A}_i)$, respectively. Then

$$\begin{aligned} \langle \varphi _0^t, \varphi _1^t, \dots , \varphi _n^t \rangle _{(\omega _0, \dots , \omega _n)} = \langle {\varphi '}_0^t, \varphi _1^t, \dots , \varphi _n^t \rangle _{(\omega _0, \dots , \omega _n)} + O(1) \end{aligned}$$

as $$t \rightarrow +\infty $$.

#### Proof

For each *i*, $0 \leqslant i \leqslant n$, we have $\varphi _i^t={\varphi '}_i^t+O(1)$ as $t\rightarrow +\infty$. Recall that the mass of the Bedford–Taylor product $\bigwedge (\omega _i + dd^c\varphi _i^t)$ is computed in cohomology, thus independent of *t*. Hence, the quantity

$$\begin{aligned}&\left\langle \varphi _0^t, \varphi _1^t, \dots , \varphi _n^t \right\rangle _{(\omega _0, \dots , \omega _n)} - \left\langle {\varphi '}_0^t, \varphi _1^t, \dots , \varphi _n^t \right\rangle _{(\omega _0, \dots , \omega _n)}\\&\quad = \int _X \left( \varphi _0^t - {\varphi '}_0^t \right) \left( \omega _1 + dd^c\varphi _1^t \right) \wedge \dots \wedge \left( \omega _n + dd^c\varphi _n^t \right) \end{aligned}$$

is bounded as $t \rightarrow +\infty$. By symmetry, the argument may be repeated for the remaining *i*, yielding the result. $\square$

### Asymptotic Slope of Deligne Functionals: Proof of Theorem B

With the above formalism in place, we are ready to formulate the main result of this section (Theorem B of the introduction). It constitutes the main contribution towards establishing Theorem A, and may be viewed as a transcendental analogue of Lemma 4.3 in [13]. We here formulate and prove the theorem in the ‘smooth but not necessarily Kähler’ setting (see Sect. 4.1, situation (1)). However, one should note that there is also a valid formulation for $L^{\infty }$-compatible subgeodesics, as pointed out in Remark 4.11.

#### Theorem 4.9

Let *X* be a compact Kähler manifold of dimension *n* and let $$\theta _i$$, $$0 \leqslant i \leqslant n$$, be closed (1, 1)-forms on *X*. Set $$\alpha _i := [\theta _i] \in H^{1,1}(X,\mathbb {R})$$. Consider smooth cohomological test configurations $$(\mathcal {X}_i, \mathcal {A}_i)$$ for $$(X,\alpha _i)$$ dominating $$X \times \mathbb {P}^1$$. For each collection of smooth rays $$(\varphi _i^t)_{t \geqslant 0}$$$$\mathcal {C}^{\infty }$$-compatible with $$(\mathcal {X}_i, \mathcal {A}_i)$$, respectively, the asymptotic slope of the multivariate energy functional $$\langle \cdot , \dots , \cdot \rangle := \langle \cdot , \dots , \cdot \rangle _{(\theta _0, \dots , \theta _n)}$$ is well defined and satisfies

$$\begin{aligned} \frac{\left\langle \varphi _0^t, \dots , \varphi _n^t \right\rangle }{t} \longrightarrow (\mathcal {A}_0 \cdot \dots \cdot \mathcal {A}_n) \end{aligned}$$

as $t \rightarrow +\infty$. See Sect. 4.1 for the definition of the above intersection number in case the $\mathcal {X}_i$ are not all equal.

#### Proof

Fix any smooth $S^1$-invariant (1, 1)-forms $\Omega _i$ on $\mathcal {X}_i$ such that $[\Omega _i] =\mathcal {A}_i$ in $H^{1,1}(\mathcal {X}_i, \mathbb {R})$. Let $(\varphi _i^t)_{t\geqslant 0}$ be smooth and $\mathcal {C}^{\infty }$-compatible with $(\mathcal {X}_i,\mathcal {A}_i)$, respectively. Let $\mathcal {X}$ be a smooth test configuration that simultaneously dominates the $\mathcal {X}_i$. By pulling back to $\mathcal {X}$ we can assume that the $\mathcal {X}_i$ are all equal (note that the notion of being $\mathcal {C}^{\infty }$-compatible is preserved under this pullback).

In the notation of Sect. 4.1, the functions $\Phi _i\circ \mu +\psi _D$ are then smooth on the manifold with boundary $M:=\pi ^{-1}(\bar{\Delta })$, and may thus be written as the restriction of smooth $S^1$-invariant functions $\Psi _i$ on $\mathcal {X}$, respectively.

Using the $\mathbb {C}^*$-equivariant isomorphism $\mathcal {X}{\setminus }\mathcal {X}_0\simeq X\times (\mathbb {P}^1{\setminus }\{0\})$ we view $(\Psi _i-\psi _D)_{| \mathcal {X}_{\tau }}$ as a function $\varphi _i^{\tau }\in \mathcal {C}^{\infty }(X)$. By Proposition 2.8 we then have $\square$

#### Lemma 4.10

Over $\mathbb {P}^1{\setminus }\{0\}$ we have

$$\begin{aligned} dd^c_{\tau } \left\langle \varphi _0^t, \dots , \varphi _n^t \right\rangle =\pi _*\left( \bigwedge _i (\Omega _i + dd^c\Psi _i)\right) . \end{aligned}$$

#### Proof

The result follows from Proposition 2.8 and the fact that $\mu$ is a biholomorphism away from $\tau = 0$, where also $\delta _D = 0$ (recalling that the $\mathbb {R}$-divisor *D* is supported on $\mathcal {X}_0$). $\square$

Denoting by $u(\tau ) := \langle \varphi _0^{\tau }, \ldots , \varphi _n^{\tau } \rangle$, the Green–Riesz formula then yields

$$\begin{aligned} \frac{d}{dt}_{t=-\log \varepsilon } u(\tau )= & {} \int _{\mathbb {P}^1{\setminus }\Delta _{\varepsilon }} dd^c_{\tau } u(\tau )\\= & {} \int _{\pi ^{-1}(\mathbb {P}^1{\setminus }\Delta _{\varepsilon })}\bigwedge _i (\Omega _i + dd^c\Psi _i), \end{aligned}$$

which converges to $(\mathcal {A}_0 \cdot \dots \cdot \mathcal {A}_n)$ as $\varepsilon \rightarrow 0$.

It remains to show that

$$\begin{aligned} \lim _{t \rightarrow +\infty } \frac{ u(\tau )}{t} = \lim _{t \rightarrow +\infty } \frac{d}{dt} u(\tau ). \end{aligned}$$

To see this, note that for each closed (1, 1)-form $\Theta$ on $\mathcal {X}$ and each smooth function $\Phi$ on $\mathcal {X}$, there is a Kähler form $\eta$ on $\mathcal {X}$ and a constant *C* large enough so that $\Theta + C\eta + dd^c\Phi \geqslant 0$ on $\mathcal {X}$. Moreover, we have a relation

$$\begin{aligned} \langle \varphi _0^t, \varphi _1^t, \dots , \varphi _n^t \rangle _{(\omega - \omega ', \theta _1 \dots , \theta _n)} =\langle {\varphi _0^t}, \varphi _1^t \dots , \varphi _n^t \rangle _{(\omega , \theta _1, \dots , \theta _n)} - \langle 0, \varphi _1^t \dots , \varphi _n^t \rangle _{(\omega ', \theta _1, \dots , \theta _n)} \end{aligned}$$

and repeat this argument for each *i*, $0 \leqslant i \leqslant n$, by symmetry. It follows from the above ‘multilinearity’ that we can write $t \mapsto E(\varphi _0^t, \dots , \varphi _n^t)$ as a difference of convex functions, concluding the proof. $\square$

#### Remark 4.11

The above proof in fact also yields a version of Theorem 4.9 for *subgeodesics*$(\varphi _i^t)_{t \geqslant 0}$ that are $L^{\infty }$*-compatible* with smooth test configurations $(\mathcal {X}_i, \mathcal {A}_i)$ for $(X,\alpha _i)$ dominating $X \times \mathbb {P}^1$. This follows from the observation that one may replace $L^{\infty }$-compatible subgeodesic rays with smooth $\mathcal {C}^{\infty }$-compatible ones, using Lemmas 4.4 and 4.8.

As a special case of Theorem B we obtain transcendental versions of several previously known formulas (see for instance [13]). As an example, we may deduce the following formula for the asymptotics of the Monge–Ampère energy functional by recalling that if $\omega$ is a Kähler form on *X* and $(\varphi _t)_{t \geqslant 0}$ is a subgeodesic ray, then

$$\begin{aligned} \mathrm {E}(\varphi _t) = \frac{1}{(n+1)V} \langle \varphi _t, \dots , \varphi _t \rangle _{(\omega , \dots , \omega )}. \end{aligned}$$

#### Corollary 4.12

Assume that $$(\mathcal {X},\mathcal {A})$$ is smooth and dominates $$X\times \mathbb {P}^1$$. For each smooth ray $$(\varphi _t)_{t \geqslant 0}$$$$\mathcal {C}^{\infty }$$-compatible with $$(\mathcal {X},\mathcal {A})$$, we then have

$$\begin{aligned} \lim _{t\rightarrow +\infty }\frac{\mathrm {E}(\varphi _t)}{t}=\mathrm {E}^{\mathrm {NA}}(\mathcal {X},\mathcal {A}) \end{aligned}$$

with

$$\begin{aligned} \mathrm {E}^{\mathrm {NA}}(\mathcal {X},\mathcal {A}):=\frac{(\mathcal {A}^{n+1})}{(n+1)V}. \end{aligned}$$

#### Remark 4.13

Here $\mathrm {E}^{\mathrm {NA}}$ makes reference to the *non-Archimedean Monge–Ampère energy functional*, see [12] for an explanation of the terminology.

To give a second example of an immediate corollary, interesting in its own right, we state the following (compare [25]):

#### Corollary 4.14

Assume that $$(\mathcal {X},\mathcal {A})$$ is smooth and dominates $$X\times \mathbb {P}^1$$. For each smooth ray $$(\varphi _t)_{t \geqslant 0}$$$$\mathcal {C}^{\infty }$$-compatible with $$(\mathcal {X},\mathcal {A})$$, we then have

$$\begin{aligned} \lim _{t\rightarrow +\infty }\frac{\mathrm {J}(\varphi _t)}{t}= \mathrm {J}^{\mathrm {NA}}(\mathcal {X}, \mathcal {A}), \end{aligned}$$

where

$$\begin{aligned} \mathrm {J}^{\mathrm {NA}}(\mathcal {X},\mathcal {A}) := \frac{(\mathcal {A} \cdot \mu ^*p_1^*\alpha ^n)}{V} - \mathrm {E}^{\mathrm {NA}}(\mathcal {X},\mathcal {A}). \end{aligned}$$

#### Proof

Note that we may write $\mathrm {J}(\varphi _t) = V^{-1}\langle \varphi _t, 0,\dots ,0 \rangle _{(\omega , \dots , \omega )} - \mathrm {E}(\varphi _t)$ and apply Theorem 4.9. $\square$

## Asymptotics for the K-Energy

Let $(X,\omega )$ be a compact Kähler manifold and $\alpha := [\omega ] \in H^{1,1}(X,\mathbb {R})$ a Kähler class on *X*. As before, let $(\mathcal {X}, \mathcal {A})$ be a smooth, relatively Kähler cohomological test configuration for $(X, \alpha )$ dominating $X \times \mathbb {P}^1$. In this section we explain how the above Theorem 4.9 can be used to compute the asymptotic slope of the Mabuchi (K-energy) functional along rays $(\varphi ^t)$, $\mathcal {C}^{1,\bar{1}}$-compatible with $(\mathcal {X},\mathcal {A})$. It is useful to keep the case of weak geodesic rays (as constructed in Lemma 4.6) in mind, which in turn implies K-semistability of $(X,\alpha )$ (Theorem A).

Regarding the proof of Theorem C, we will see that the Mabuchi functional is in fact of the form $\langle \varphi _0^t, \dots , \varphi _n^t \rangle _{(\theta _0, \dots , \theta _n)}$ for the appropriate choice of closed (1, 1)-forms $\theta _i$ on *X* and rays $(\varphi _i^t)$ on *X*, but Theorem 4.9 does not directly apply in this situation. Indeed, the expression for the Mabuchi functional involves the metric $\log (\omega + dd^c\varphi _t)^n$ on $K_{\mathcal {X} / \mathbb {P}^1}$, which may blow up close to $\mathcal {X}_0$ (in particular, the compatibility conditions are not satisfied). However, a key point is that we can cook up a functional $\mathrm {M}_{\mathcal {B}}$ of the above ‘multivariate’ form that satisfies the same asymptotic slope as the Mabuchi functional (up to an explicit error term), and to which we may apply Theorem 4.9. More precisely, we show that

$$\begin{aligned} \lim _{t \rightarrow +\infty } \frac{\mathrm {M}(\varphi _{t})}{t} = \lim _{t \rightarrow +\infty } \frac{\mathrm {M}_{\mathcal {B}}(\varphi _{t})}{t} + V^{-1}((\mathcal {X}_{0,\mathrm {red}} - \mathcal {X}_0) \cdot \mathcal {A}^n)_{\mathcal {X}}, \end{aligned}$$

and use Theorem 4.9 to choose $\mathrm {M}_{\mathcal {B}}$ so that moreover $\lim _{t \rightarrow +\infty } \mathrm {M}_{\mathcal {B}}(\varphi _t)/t = \mathrm {DF}(\mathcal {X},\mathcal {A})$. It follows that the asymptotic slope of the Mabuchi (K-energy) functional equals $\mathrm {DF}(\mathcal {X}, \mathcal {A}) + V^{-1}((\mathcal {X}_{0,\mathrm {red}} - \mathcal {X}_0) \cdot \mathcal {A}^n)_{\mathcal {X}} =: \mathrm {M}^{\mathrm {NA}}(\mathcal {X}, \mathcal {A})$.

### A Weak Version of Theorem C

We first explain how to obtain a weak version of Theorem C, as a direct consequence of Theorem 4.9. This version is more direct to establish than the full Theorem C, and will in fact be sufficient in order to prove K-semistability of $(X,\alpha )$, as explained in Sect. 5.2.

#### Theorem 5.1

Let $(\mathcal {X}, \mathcal {A})$ be a smooth, relatively Kähler cohomological test configuration for $(X,\alpha )$ dominating $X \times \mathbb {P}^1$. For each subgeodesic ray $(\varphi ^t)_{t \geqslant 0}$, $\mathcal {C}^{1,\bar{1}}$-compatible with $(\mathcal {X},\mathcal {A})$, we have the inequality^3^

$$\begin{aligned} \overline{\lim }_{t \rightarrow +\infty } \frac{\mathrm {M}(\varphi _{t})}{t} \leqslant \mathrm {DF}(\mathcal {X}, \mathcal {A}). \end{aligned}$$

#### Remark 5.2

In view of the strong version (see Theorem 5.6) we actually know that the limit is well defined and, moreover, we obtain this way the precise asymptotic slope of the Mabuchi functional, see Sect. 5.3.

#### Proof of Theorem 5.1

We use additive notation for line bundles and metrics, meaning that a Hermitian metric ||.|| on a line bundle is $L \rightarrow X$ is represented by a collection of local functions $\phi := \{\phi _U\}$, defined as follows: If $U \subset X$ is an open subset and $s_U$ is a trivialising section of *L* (i.e. a local generator of the invertible sheaf $\mathcal {O}(L)$), then we set

$$\begin{aligned} \phi _U := -\log ||s_U||^2. \end{aligned}$$

Here $\phi _U$ depends on $s_U$, but the curvature current $dd^c\phi$ is globally well defined and represents the first Chern class $c_1(L)$. In the sequel we identify the additive object $\phi$ with the Hermitian metric it represents.

In the above sense, now let $\mathcal {B}$ be any smooth metric on $K_{ \mathcal {X} / \mathbb {P}^1} := K_{\mathcal {X}} - \pi ^*K_{\mathbb {P}^1}$. Consider the canonical isomorphism $\mu : \mathcal {X} {\setminus } \mathcal {X}_0 \rightarrow X \times (\mathbb {P}^1 {\setminus } \{0\})$. Since the restriction of $K_{\mathcal {X}/ \mathbb {P}^1}$ to each fibre $\mathcal {X}_t$ coincides with $K_{\mathcal {X}_t}$, which in turn can be identified with $K_X$ via $\mu$, we can then associate with $\mathcal {B}$ a ray of smooth metrics on $K_X$ that we denote by $(\beta _t)_{t \geqslant 0}$ (or $(\beta _{\tau })_{\tau \in \bar{\Delta }^*}$ for its reparametrisation by $t = -\log |\tau |$). Fix $\log \omega ^n$ as a reference metric on $K_X$, and let

7 $$\begin{aligned} \xi _{\mathcal {B}}^t := \log \left( \frac{e^{\beta _{\tau }}}{\omega ^n} \right) , \end{aligned}$$,

i.e. the function on *X* given as the difference of metrics $\beta _{\tau } - \log \omega ^n$ on $K_X$. The constructed ray $(\xi _{\mathcal {B}}^t)_{t \geqslant 0}$ is then $\mathcal {C}^{\infty }$-compatible with the cohomological test configuration $(\mathcal {X}, K_{\mathcal {X} / \mathbb {P}^1})$ for $(X,K_X)$.

Now let $(\varphi _t)_{t \geqslant 0}$ be any subgeodesic ray $\mathcal {C}^{1,\bar{1}}$-compatible with $(\mathcal {X}, \mathcal {A})$. By Lemmas 4.4, 4.8 and Theorem 4.9 it follows that

8 $$\begin{aligned} \frac{ \langle \xi _{\mathcal {B}}^t, \varphi _t \dots , \varphi _t \rangle _{(-\text {Ric}(\omega ), \omega \dots , \omega )}}{t} \longrightarrow (K_{\mathcal {X}/\mathbb {P}^1} \cdot \mathcal {A}^n)_{\mathcal {X}} \end{aligned}$$

as $t \rightarrow +\infty$. Indeed, by Lemma 4.4 we may choose a smooth subgeodesic ray $(\varphi '_t)_{t\geqslant 0}$ in $\mathcal {H}$ that is $\mathcal {C}^{\infty }$-compatible (and hence also $L^{\infty }$- and $\mathcal {C}^{1,\bar{1}}$-compatible) with $(\mathcal {X},\mathcal {A})$. Up to replacing $(\varphi _t)$ with $(\varphi '_t)$ we may thus assume that $(\varphi _t)$ is smooth and $\mathcal {C}^{\infty }$-compatible with $(\mathcal {X},\mathcal {A})$, using Lemma 4.8, so that Theorem 4.9 applies.

Motivated by the Chen–Tian formula (5) and the identity (8), we thus introduce the notation

$$\begin{aligned} \mathrm {M}_{\mathcal {B}}(\varphi _t) := \bar{\mathcal {S}} \mathrm {E}(\varphi _{\tau }) + V^{-1} \langle \xi _{\mathcal {B}}^t, \varphi _t \dots , \varphi _t \rangle _{(-\text {Ric}(\omega ), \omega \dots , \omega )}, \end{aligned}$$

the point being that the asymptotic slope of this functional coincides with the Donaldson–Futaki invariant (even when the central fibre is not reduced). $\square$

#### Lemma 5.3

$$\begin{aligned} \lim _{t \rightarrow +\infty } \frac{\mathrm {M}_{\mathcal {B}}(\varphi _t)}{t} = \mathrm {DF}(\mathcal {X}, \mathcal {A}). \end{aligned}$$

#### Proof

This result is an immediate consequence of (8), the Chen–Tian formula (5) and Corollary 4.12. $\square$

Hence, it suffices to establish the following inequality

$$\begin{aligned} \overline{\lim }_{t \rightarrow +\infty } \frac{\mathrm {M}(\varphi _t)}{t} \leqslant \lim _{t \rightarrow +\infty } \frac{\mathrm {M}_{\mathcal {B}}(\varphi _t)}{t}. \end{aligned}$$

To do this, we set $\Gamma (\tau ) := (\mathrm {M} - \mathrm {M}_{\mathcal {B}})(\varphi _t).$ By the Chen–Tian formula (5) and cancellation of terms we have

$$\begin{aligned} \Gamma (\tau )= & {} \bar{\mathcal {S}} \mathrm {E}(\varphi _t) - \mathrm {E}^{\text {Ric}(\omega )}(\varphi _t) + V^{-1} \int _X \log \left( \frac{(\omega + dd^c\varphi _{\tau })^n}{\omega ^n} \right) (\omega + dd^c\varphi _{\tau })^n\\&- \bar{\mathcal {S}} \mathrm {E}(\varphi _t) - V^{-1} \langle \xi _{\mathcal {B}}^t, \varphi _t \dots , \varphi _t \rangle _{(-\text {Ric}(\omega ), \omega \dots , \omega )}\\= & {} - \mathrm {E}^{\text {Ric}(\omega )}(\varphi _t) + V^{-1} \int _X \log \left( \frac{(\omega + dd^c\varphi _{\tau })^n}{\omega ^n} \right) (\omega + dd^c\varphi _{\tau })^n \\&- V^{-1} \int _X \xi _{\mathcal {B}}^t \; (\omega + dd^c\varphi _{\tau })^n\\&+ V^{-1} \sum _{j = 0}^{n-1} \int _X \varphi _t \; \text {Ric}(\omega ) \wedge \omega ^j \wedge (\omega + dd^c\varphi _t)^{n-j-1}\\= & {} V^{-1} \int _X \log \left( \frac{(\omega + dd^c\varphi _{\tau })^n}{\omega ^n} \right) (\omega + dd^c\varphi _{\tau })^n \\&- V^{-1} \int _X \log \left( \frac{e^{\beta _{\tau }}}{\omega ^n} \right) \; (\omega + dd^c\varphi _{\tau })^n\\= & {} V^{-1} \int _X \log \left( \frac{(\omega + dd^c\varphi _{\tau })^n}{e^{\beta _{\tau }}} \right) (\omega + dd^c\varphi _{\tau })^n, \end{aligned}$$

recalling the definition (7) of $\xi _{\mathcal {B}}^t$ and Definition 2.1.

In view of Proposition 3.10, we as usual let *D* denote the unique $\mathbb {R}$-divisor supported on $\mathcal {X}_0$ such that $\mathcal {A}=\mu ^*p_1^*\alpha +[D],$ with $p_1:X \times \mathbb {P}^1 \rightarrow X$ the first projection. Fix a choice of an $S^1$-invariant function ‘Green function’ $\psi _D$ for *D*, so that $\delta _D=\theta _D+dd^c\psi _D$ with $\theta _D$ a smooth $S^1$-invariant closed (1, 1)-form on $\mathcal {X}$. Moreover, set $\Omega := \mu ^*p_1^*\alpha + \theta _D$ (for which $[\Omega ] = \mathcal {A}$ then holds) and let $\Phi$ denote the $S^1$-invariant function on $X \times \mathbb {P}^1$ corresponding to the ray $(\varphi _t)$. In particular, the function $\Phi \circ \mu + \psi _D$ extends to a smooth $\Omega$-psh function $\Psi$ on $\mathcal {X}$, by $\mathcal {C}^{\infty }$-compatibility.

With the above notation in place, the integrand in the above expression for $\Gamma (\tau )$ can be written as

$$\begin{aligned} \log \left( \frac{(\omega + dd^c\varphi _{\tau })^n}{e^{\beta _{\tau }}} \right) = \mu _* \left( \log \left( \frac{(\Omega + dd^c\Psi )^n \wedge \pi ^*(\sqrt{-1} \; d\tau \wedge d\bar{\tau })}{\lambda _{\mathcal {B}}} \right) \right) , \end{aligned}$$

where

$$\begin{aligned} \lambda _{\mathcal {B}} := e^{\mathcal {B} + \pi ^*\log (\sqrt{-1} \; d\tau \wedge d\bar{\tau })} \end{aligned}$$

is the volume form defined by the smooth metric $\mathcal {B} + \pi ^*\log (\sqrt{-1} \; d\tau \wedge d\bar{\tau })$ on $K_{\mathcal {X}}$. Since $\Psi$ is smooth on $\mathcal {X}$ and $\lambda _{\mathcal {B}}$ is a volume form on $\mathcal {X}$, this quantity is bounded from above. Moreover, we integrate against the measure $(\omega + dd^c\varphi _{\tau })^n$ which can be computed in cohomology, and thus has mass independent of $\tau$. Hence

$$\begin{aligned} \Gamma (\tau ) = V^{-1} \int _X \log \left( \frac{(\omega + dd^c\varphi _{\tau })^n}{e^{\beta _{\tau }}} \right) (\omega + dd^c\varphi _{\tau })^n \leqslant O(1). \end{aligned}$$

Dividing by *t* and passing to the limit now concludes the proof. $\square$

As explained below, the above ‘weak Theorem C’ actually suffices to yield our main result.

### Proof of Theorem A

We now explain how the above considerations apply to give a proof of Theorem A and point out some immediate and important consequences regarding the YTD conjecture. First recall the following definition (see, e.g. [51, Sect. 7.2]):

#### Definition 5.4

We say that $(X, \alpha )$ is *uniformly K-stable* if there is a $\delta > 0$ and $C \geqslant 0$ such that

$$\begin{aligned} \mathrm {M}^{\mathrm {NA}}(\mathcal {X}, \mathcal {A}) \geqslant \delta \mathrm {J}^{\mathrm {NA}}(\mathcal {X}, \mathcal {A}) - C \end{aligned}$$

for all relatively Kähler cohomological test configurations $(\mathcal {X}, \mathcal {A})$ for $(X,\alpha )$.

We are now ready to prove Theorem A.

#### Proof of Theorem A

Let *X* be a compact Kähler manifold and $\omega$ a given Kähler form, with $\alpha := [\omega ] \in H^{1,1}(X,\mathbb {R})$ the corresponding Kähler class. Let $(\mathcal {X},\mathcal {A})$ be any (possibly singular) cohomological test configuration for $(X,\alpha )$ which by desingularisation and perturbation (see Proposition 3.12) can be assumed to be smooth, relatively Kähler and dominating $X \times \mathbb {P}^1$. Consider any ray $(\varphi _t)_{t \geqslant 0}$ such that Theorem C applies; for instance, one may take $(\varphi _t)$ to be the associated weak geodesic ray emanating from $\omega$ (i.e. such that $\varphi _0 = 0$), which due to [15] (cf. also [9, 20, 21]) is $\mathcal {C}^{1,\bar{1}}$-compatible with $(\mathcal {X}, \mathcal {A})$. Now suppose that the Mabuchi functional is bounded from below (in the given class $\alpha$). In particular, we then have

$$\begin{aligned} \mathrm {DF}(\mathcal {X},\mathcal {A}) \geqslant \overline{\lim }_{t \rightarrow +\infty } \frac{\mathrm {M}(\varphi _t)}{t} \geqslant 0, \end{aligned}$$

using the weak version of Theorem C, cf. Theorem 5.1. Since the cohomological test configuration $(\mathcal {X}, \mathcal {A})$ for $(X,\alpha )$ was chosen arbitrarily, this proves Corollary 1.1, i.e. it shows that $(X,\alpha )$ is K-semistable.

In a similar vein, suppose that the Mabuchi functional is coercive, i.e. in particular $\mathrm {M}(\varphi _t) \geqslant \delta \mathrm {J}(\varphi _t) - C$ for some constants $\delta , C > 0$ uniform in *t*. Note that Corollary 4.14) and the (weak) Theorem C provides a link with the intersection theoretic quantities $\mathrm {J}^{\mathrm {NA}}(\mathcal {X}, \mathcal {A})$ and $\mathrm {M}^{\mathrm {NA}}(\mathcal {X}, \mathcal {A})$, respectively. More precisely, dividing by *t* and passing to the limit we have

$$\begin{aligned} 0 \leqslant \overline{\lim }_{t \rightarrow +\infty } \frac{(\mathrm {M} - \delta \mathrm {J})(\varphi _t)}{t} \leqslant \mathrm {M}^{\mathrm {NA}}(\mathcal {X}, \mathcal {A}) - \delta \mathrm {J}^{\mathrm {NA}}(\mathcal {X}, \mathcal {A}). \end{aligned}$$

Since $(\mathcal {X}, \mathcal {A})$ was chosen arbitrarily it follows that $(X,\alpha )$ is uniformly K-stable, concluding the proof of Theorem A. $\square$

As remarked in the introduction it follows from convexity of the Mabuchi functional along weak geodesic rays, cf. [6, 19], that the Mabuchi functional is bounded from below (in the given class $\alpha$) if $\alpha$ contains a cscK representative. In other words, Corollary 1.1 follows.

Moreover, it is shown in [8, Theorem 1.2] that the Mabuchi functional $\mathrm {M}$ is in fact coercive if $\alpha$ contains a cscK representative. As a consequence, we obtain also the following stronger result, confirming the “if” direction of the YTD conjecture (here referring to its natural generalisation to the transcendental setting, using the notions introduced in Sect. 3).

#### Corollary 5.5

If the Kähler class $\alpha \in H^{1,1}(X,\mathbb {R})$ admits a constant scalar curvature representative, then $(X,\alpha )$ is uniformly K-stable.

### Asymptotic Slope of the K-Energy

Building on Sect. 5.1 we now improve on the weak version of Theorem C (cf. Theorem 5.1) by computing the asymptotic slope of the Mabuchi (K-energy) functional (even when the central fibre is not reduced). To this end, recall the definition of the non-Archimedean Mabuchi functional, i.e. the intersection number

$$\begin{aligned} \mathrm {M}^{\mathrm {NA}}(\mathcal {X}, \mathcal {A}) := \mathrm {DF}(\mathcal {X}, \mathcal {A}) + V^{-1}((\mathcal {X}_{0,\mathrm {red}} - \mathcal {X}_0) \cdot \mathcal {A}^n)_{\mathcal {X}}, \end{aligned}$$

discussed in Sect. 3.6. Note that it satisfies $\mathrm {M}^{\mathrm {NA}}(\mathcal {X}, \mathcal {A}) \leqslant \mathrm {DF}(\mathcal {X}, \mathcal {A})$ with equality precisely when the central fibre is reduced.

Adapting the techniques of [13] to the present setting we now obtain the following result, corresponding to Theorem C of the introduction.

#### Theorem 5.6

Let *X* be a compact Kähler manifold and $\alpha \in H^{1,1}(X,\mathbb {R})$ a Kähler class. Suppose that $(\mathcal {X}, \mathcal {A})$ is a smooth, relatively Kähler cohomological test configuration for $(X,\alpha )$ dominating $X \times \mathbb {P}^1$. Then, for each subgeodesic ray $(\varphi _t)_{t \geqslant 0}$, $\mathcal {C}^{1,\bar{1}}$-compatible with $(\mathcal {X},\mathcal {A})$, the asymptotic slope of the Mabuchi functional is well defined and satisfies

$$\begin{aligned} \frac{\mathrm {M}(\varphi _{t})}{t} \longrightarrow \mathrm {M}^{\mathrm {NA}}(\mathcal {X}, \mathcal {A}) \end{aligned}$$

as $$t \rightarrow +\infty $$.

#### Remark 5.7

In particular, this result holds when $(\varphi _t)_{t \geqslant 0}$ is the weak geodesic ray associated with $(\mathcal {X},\mathcal {A})$, constructed in Sect. 4.1.

#### Proof of Theorem 5.6

Following ideas of [13] we associate with the given smooth, relatively Kähler and dominating test configuration $(\mathcal {X}, \mathcal {A})$ for $(X,\alpha )$ another test configuration $(\mathcal {X}', \mathcal {A}')$ for $(X,\alpha )$ which is semistable, i.e. smooth and such that $\mathcal {X}'_0$ is a reduced $\mathbb {R}$-divisor with simple normal crossings. As previously noted, we can also assume that $\mathcal {X}'$ dominates the product. In the terminology of Sect. 3.6, this construction comes with a morphism $g_d \circ \rho : \mathcal {X}' \rightarrow \mathcal {X}$, cf. the diagram in Section 3.6. Pulling back, we set $\mathcal {A}' := g_d^*\rho ^*\mathcal {A}$. Note that $\mathcal {A}'$ is no longer relatively Kähler, but merely relatively semipositive (with the loss of positivity occurring along $\mathcal {X}_0'$).

On the one hand, Lemma 3.16 yields

9 $$\begin{aligned} \mathrm {M}^{\mathrm {NA}}(\mathcal {X}', \mathcal {A}') = d \cdot \mathrm {M}^{\mathrm {NA}}(\mathcal {X}, \mathcal {A}), \end{aligned}$$

where $d > 0$ is the degree of the morphism $g_d$. On the other hand, we may consider the pullback by $g_d \circ \rho$ of the weak geodesic $(\varphi _t)_{t \geqslant 0}$ associated with $(\mathcal {X}, \mathcal {A})$. This induces a subgeodesic $({\varphi }_t')_{t \geqslant 0}$ which is $\mathcal {C}^{1,\bar{1}}$-compatible with the test configuration $(\mathcal {X}', \mathcal {A}')$ for $(X, \alpha )$ (in particular, the boundedness of the Laplacian is preserved under pullback by $g_d \circ \rho$). Replacing $\tau$ by $\tau ^d$ amounts to replacing *t* by $d \cdot t$, so that

10 $$\begin{aligned} \frac{\mathrm {M}({\varphi }_t')}{t} = d \cdot \frac{\mathrm {M}(\varphi _t)}{t}. \end{aligned}$$

Combining equations (9) and (10) it thus follows that

$$\begin{aligned} \lim _{t \rightarrow +\infty }\frac{\mathrm {M}(\varphi _{t})}{t} = \mathrm {M}^{\mathrm {NA}}(\mathcal {X}, \mathcal {A}) \end{aligned}$$

if and only if

11 $$\begin{aligned} \lim _{t \rightarrow +\infty }\frac{\mathrm {M}({\varphi }_{t}')}{t} = \mathrm {DF}(\mathcal {X}',\mathcal {A}'). \end{aligned}$$

In other words, it suffices to establish (11). By the asymptotic formula 5.3 it is in turn equivalent to show that

$$\begin{aligned} \lim _{t \rightarrow +\infty } \frac{\mathrm {M}({\varphi }_{t}')}{t} = \lim _{t \rightarrow +\infty } \frac{\mathrm {M}_{\mathcal {B}}({\varphi }_{t}')}{t}. \end{aligned}$$

We use the notation of the proof of Theorem 5.1. In particular, we set $\Gamma (\tau ) := (\mathrm {M} - \mathrm {M}_{\mathcal {B}})(\varphi _{\tau }')$. As in the proof of Theorem 5.1 we have an upper bound $\Gamma (\tau ) \leqslant O(1)$, using that the restriction of the relatively semipositive class $\mathcal {A}'$ to $\mathcal {X}'{\setminus } \mathcal {X}_0'$ is in fact relatively Kähler.

To obtain a lower estimate of $\Gamma (\tau )$ we consider the Monge–Ampere measure ${\mathrm {MA}}(\varphi _{\tau }') := V^{-1} (\omega + dd^c\varphi _{\tau }')^n$ and note that

$$\begin{aligned} V^{-1} \Gamma (\tau )= & {} V^{-1} \int _X \log \left( \frac{(\omega + dd^c\varphi _{\tau }')^n}{e^{\beta _{\tau }}} \right) \\= & {} \int _X \log \left( \frac{\mathrm {MA}(\varphi _{\tau }')}{e^{\beta _{\tau }} \big / \int _X e^{\beta _{\tau }}} \right) \mathrm {MA}(\varphi _{\tau }') - \log \int _X e^{\beta _{\tau }} \geqslant - \log \int _X e^{\beta _{\tau }}, \end{aligned}$$

since the relative entropy of the two probability measures $\mathrm {MA}(\varphi _{\tau })$ and $e^{\beta _{\tau }} / \int _X e^{\beta _{\tau }}$ is non-negative. We now conclude by estimating this integral, using the following result from [13]: $\square$

#### Lemma 5.8

([13]). Let $(\mathcal {X}, \mathcal {A})$ be a semistable and dominating test configuration for $(X,\alpha )$ and let $\mathcal {B}$ be any smooth metric on $K_{ \mathcal {X}/ \mathbb {P}^1}$. Let $(\beta ^t)_{t \geqslant 0}$ be the family of smooth metrics on $K_X$ induced by $\mathcal {B}$. Denote by $p \geqslant 1$ the largest integer such that $p-1$ distinct irreducible components of $\mathcal {X}_0$ have a non-empty intersection. Then there are positive constants *A* and *B* such that

$$\begin{aligned} A t^{2(p-1)} \leqslant \int _X e^{\beta ^t} \leqslant B t^{2(p-1)} \end{aligned}$$

holds for all *t*.

We refer the reader to [13] for the proof and here simply apply the result: Recalling that $t = -\log |\tau |$, Lemma 5.8 yields that $\log \int _X e^{\beta _{\tau }} = o(t)$ and so it follows from

$$\begin{aligned} \frac{-\log \int _X e^{\beta _{\tau }}}{t} \leqslant \frac{\Gamma (\tau )}{t} \leqslant \frac{O(1)}{t} \end{aligned}$$

that

$$\begin{aligned} \lim _{t \rightarrow +\infty } \frac{\Gamma (\tau )}{t} = 0, \end{aligned}$$

completing the proof. $\square$

## Footnotes

1 We use the word ‘transcendental’ to emphasise that the Kähler class in question is not necessarily of the form $c_1(L)$ for some ample line bundle *L* over *X*.

2 By a standard abuse of notation we identify a Hermitian metric *h* with its associated (1, 1)-form $\omega = \omega _h$ and refer to $\omega$ as the “metric”.

3 The limit is in fact well defined, as shown in Sect. 5.3.

## References

[1] Barth, W.P., Hulek, K., Peters, C.A.M., Van de Ven, A.: Compact Complex Surfaces, 2nd edn, vol. 4. Ergebnisse der Mathematik und ihrer Grenzgebiete. A Series of Modern Surveys in Mathematics. Springer, Berlin (2004)

[2] Bedford E, Taylor BA (1976). The Dirichlet problem for the complex Monge–Ampère operator. Invent. Math..

[3] Bedford E, Taylor BA (1982). A new capacity for plurisubharmonic functions. Acta Math..

[4] Berman, R.: From Monge–Ampère equations to envelopes and geodesic rays in the zero temperature limit. Preprint arXiv: 1307.3008 (2013)

[5] Berman R (2016). K-polystability of Q-Fano varieties admitting Kähler–Einstein metrics. Invent. Math..

[6] Berman, R., Berndtsson, B.: Convexity of the K-energy on the space of Kähler metrics and uniqueness of extremal metrics. Preprint arXiv:1405.0401 (2014)

[7] Berman R, Boucksom S, Guedj V, Zeriahi A (2013). A variational approach to complex Monge–Ampère equations. Publ. Math. de l’IHES.

[8] Berman, R., Darvas, T., Lu, C.: Regularity of weak minimizers of the K-energy and applications to properness and K-stability. arXiv:1602.03114v1 (2016)

[9] Blocki Z (2013). The Calabi–Yau Theorem. Complex Monge–Ampère Equations and Geodesics in the Space of Kähler Metrics.

[10] Boucksom S, Guedj V (2012). Monge–Ampère equations on complex manifolds with boundary. Complex Monge–Ampère Equations and Geodesics in the Space of Kähler Metrics.

[11] Boucksom S, Guedj V, Boucksom S, Eyssidieux P, Guedj V (2013). Regularizing properties of the Kähler–Ricci flow. An Introduction to the Kähler–Ricci Flow.

[12] Boucksom, S., Hisamoto, T., Jonsson, M.: Uniform K-stability, Duistermaat–Heckman measures and singularities of pairs. Preprint arXiv:1504.06568v1 (2015)

[13] Boucksom, S., Hisamoto, T., Jonsson, M.: Uniform K-stability and asymptotics of energy functionals in Kähler geometry. Preprint arXiv:1603.01026 (2016)

[14] Chen XX (2000). On the lower bound of the Mabuchi energy and its application. Int. Math. Res. Not..

[15] Chen XX (2000). The space of Kähler metrics. J. Differ. Geom..

[16] Chen XX, Donaldson S, Sun S (2015). Kähler–Einstein metrics on Fano manifolds I: approximation of metrics with cone singularities. J. Am. Math. Soc..

[17] Chen XX, Donaldson S, Sun S (2015). Kähler–Einstein metrics on Fano manifolds II: limits with cone angle less than 2pi. J. Am. Math. Soc..

[18] Chen XX, Donaldson S, Sun S (2015). Kähler–Einstein metrics on Fano manifolds III: limits as cone angle approaches 2pi and completion of the main proof. J. Am. Math. Soc..

[19] Chen XX, Li L, Paun M (2016). Approximation of weak geodesics and subharmonicity of Mabuchi energy. Ann. Fac. Sci. Toulouse Math..

[20] Darvas, T.: Envelopes and geodesics in spaces of Kähler potentials. Preprint arXiv:1401:7318 (2014)

[21] Darvas T, Lempert L (2012). Weak geodesic rays in the space of Kähler metrics. Math. Res. Lett..

[22] Darvas T, Rubinstein YA (2017). Tian’s properness conjecture and Finsler geometry of the space of Kähler metrics. J. Am. Math. Soc..

[23] Demailly, J.-P.: Complex Analytic and Differential Geometry. Open source (2012)

[24] Dervan R (2016). Uniform stability of twisted constant scalar curvature Kähler metrics. Int. Math. Res. Not..

[25] Dervan, R., Ross, J.: K-stability for Kähler manifolds. Math. Res. Lett. **24**(3), 689–739 (2017). 10.4310/MRL.2017.v24.n3.a5

[26] Donaldson SK (1985). Anti-self-dual Yang–Mills connections over complex surfaces and stable vector bundles. Proc. Lond. Math. Soc..

[27] Donaldson SK (2002). Scalar curvature and stability of toric varieties. J. Differ. Geom.

[28] Donaldson SK (2005). Lower bounds on the Calabi functional. J. Differ. Geom..

[29] Donaldson SK (2005). Scalar curvature and projective embeddings. II. Q. J. Math..

[30] Elkik R (1990). Métriques sur les fibrés d’intersection. Duke Math. J..

[31] Fischer G (1976). Complex Analytic Geometry.

[32] Kempf G, Knudsen F, Mumford D, Saint-Donat B (1973). Toroidal Embeddings 1.

[33] Kollár J (2007). Lectures on Resolution of Singularities.

[34] Kollár, J., Nicaise, J., Xu, C.Y.: Semi-stable extensions over 1-dimensional bases. Acta. Math. Sin.-English Ser. (2017). 10.1007/s10114-017-7048-8

[35] Li C (2011). Constant scalar curvature Kähler metric obtains the minimum of the K-energy. Int. Math. Res. Not..

[36] Li C, Xu C (2014). Special test configurations and K-stability of Fano varieties. Ann. Math..

[37] Mabuchi, T.: K-stability of constant scalar curvature polarization. arXiv:0812.4093 (2008)

[38] Moriwaki A (1999). The continuity of Deligne’s pairing. Int. Math. Res. Not..

[39] Odaka Y (2013). A generalization of the Ross Thomas slope theory. Osaka J. Math..

[40] Paul, S., Tian, G.: CM stability and the generalized Futaki invariant I. arXiv: math.AG/0605278 (2006)

[41] Paul S, Tian G (2009). CM stability and the generalized Futaki invariant II. Astérisque.

[42] Phong DH, Ross J, Sturm J (2008). Deligne pairings and the Knudsen–Mumford expansion. J. Differ. Geom..

[43] Ross J, Thomas RP (2006). An obstruction to the existence of constant scalar curvature Kähler metrics. J. Differ. Geom..

[44] Rubinstein YA, Albin P (2014). Smooth and singular Kähler–Einstein metrics. Geometric and Spectral Analysis.

[45] Semmes S (1992). Complex Monge–Ampère equations and symplectic manifolds. Am. J. Math.

[46] Stoppa J (2009). K-stability of constant scalar curvature Kähler manifolds. Adv. Math..

[47] Stoppa J (2009). Twisted constant scalar curvature Kähler metrics and Kähler slope stability. J. Differ. Geom..

[48] Szekelyhidi G (2014). Introduction to extremal Kähler metrics.

[49] Tian G (1997). Kähler–Einstein metrics with positive scalar curvature. Invent. Math..

[50] Tian G (2000). Bott–Chern forms and geometric stability. Discret. Contin. Dyn. Syst..

[51] Tian G (2000). Canonical Metrics in Kähler Geometry.

[52] Tian G (2015). K-stability and Kähler–Einstein metrics. Commun. Pure Appl. Math..

[53] Wang X (2012). Height and GIT weight. Math. Res. Lett..

[54] Yau S-T (2006). Perspectives on geometric analysis. Surv. Differ. Geom..

[55] Zhang S-W (1996). Heights and reductions of semi-stable varieties. Compos. Math..

